# Ratiometric Mass Spectrometry for Cell Identification and Quantitation Using Intracellular “Dual-Biomarkers”

**DOI:** 10.1038/s41598-017-17812-1

**Published:** 2017-12-12

**Authors:** Xiaoming Chen, Fangjie Wo, Jiang Chen, Jie Tan, Tao Wang, Xiao Liang, Jianmin Wu

**Affiliations:** 1 Institute of Analytical Chemistry, Department of Chemistry, Zhejiang University, Hangzhou, 310058 China; 2 Department of General Surgery, Sir Run Run Shaw Hospital, School of Medicine, Zhejiang University, Hangzhou, 310016 China

## Abstract

This study proposed an easy-to-use method for cell identification and quantitation by ratiometric matrix-assisted laser desorption/ionization time-of-flight mass spectrometry (MALDI-TOF MS). Two pairs of MS peaks in the molecular fingerprint of cells were selected as intracellular dual-biomarkers due to the stability and specificity of their ratio values in different types of hepatocellular cancer (HCC) cell lines. Five types of HCC cells can be thereafter differentiated based on these two pairs of intracellular peptides/proteins. Two types of HCC cells, Huh7 and LM3 were co-cultured as a model to test whether the method is feasible for cell quantitation. The results indicated that the ratiometric peak intensity of the two pair biomarkers exhibits linear relationship with the proportion of Huh7 cells. Furthermore, tumor heterogeneity was simulated by subcutaneously injecting the co-cultured cells into nude mice. The cell type and proportion in the section of grown tumor tissue can be discriminated using the ratiometric MALDI imaging approach. LC-MS/MS detection revealed that one of the biomarker pairs belongs to thymosin family, β4 and β10. The ratiometric MS spectral approach using intracellular dual-biomarkers might become a pervasive strategy for high-throughput cell identification and quantitation, which is vital in tumor heterogeneity study, clinical diagnosis and drug screening.

## Introduction

Tumor heterogeneity^1^ is one of the characteristics in malignant tumor, which commonly occurs among different individuals^2–4^. With the genetic and epigenetic influence, cells gradually show difference in molecular biology. Even in the same tumor, clonal evolution probably proceeds in a branching rather than in a linear manner^5^, resulting in phenotypic and functional heterogeneity within the tumor^6^. Phenotypic heterogeneity has been related to the clinical results such as prognosis, resistance to medicine and the capability of metastasis^7^, all of which make precision medicine^8^ more challenging. Therefore, cell identification in tumors is critical to deeply explore the inter- and intra-tumor heterogeneity and further can provide effective guides for personal diagnosis.

In the last two decades, various methods and technologies have been developed for cell identification based on chemical/biological^9–12^ and physical characteristics^13–15^ belonging to cells and tissues. Gene sequencing is an effective approach that can detect most mutations in DNA within cells^9,16^. To further provide insight into genomic diversity, a single cell sequencing approach called nuc-seq has been developed recently^17^. Besides the mutation, DNA methylation plays a crucial role in defining cell types. With the advance of reduced representation bisulfite sequencing (RRBS) technology, information of tumor specification and heterogeneity among the cell types can be obtained according to methylation patterns^18^. Gene sequencing is useful and accurate, but it is relatively expensive and time consuming and not appropriate for routine detection. On the other hand, in many cases, therapeutic resistance can be linked to altered gene expression patterns without associated changes in DNA sequence. Therefore, except for genomics study, cell identification methods based on the expressed proteins or peptides play vital roles in the study of tumor heterogeneity. Currently, flow cytometry combined with fluorescence^10^, ICP-MS imaging^19^, Raman^11^ and microfluidic technology^11,20^ have been used to reveal tumor heterogeneity according to the specificity of cell-surface receptors. However, those cells sharing similar surface receptors cannot be discriminated using flow cytometric technology. In addition, the inner cell molecular information cannot be obtained. Mass spectrometry (MS) is an ideal tool for cell analysis since MS can provide almost all molecular information in cells^21,22^. Liquid Chromatography (LC) coupled MS or MS/MS has been commercially available for intracellular proteomics and peptidomics study^23^. However, it is facing major technological challenges in achieving the goals of comprehensive, reproducible, and quantitative results of proteomes at reasonable throughput^24^. Among various MS techniques, matrix-assisted laser desorption/ionization time-of-flight mass spectrometry (MALDI-TOF MS)^25^ is a high throughput technology, which can generate cell molecular fingerprint^26^. Coupled with the MS database of microorganism, identification and classification of bacterial species with MALDI-TOF MS have been achieved^27–29^. In 1998, whole-cell detection using MALDI-TOF MS was proposed^30^, and the possibility to discriminate different mammalian lines has been demonstrated in recent researches^12,31,32^. However, the bad quantification ability of MALDI-TOF MS hinders its wide application in cell identification and tumor heterogeneity targeting precision medicine. While stable-isotope label technology helps to resolve the quantification problems^33–35^, those label strategies would increase the complexity of MS spectra. In addition, when quantification at the cellular level is required, SILAC (stable isotope labeling with amine acid in cell culture) would need 5 or 6 generation passages before cell detection by MS^36^. Compared to label strategies, label free methods recently developed are mainly depending on statistical calculation^37–40^. Therefore, an effective, sensitive and convenient method for cell identification and tumor heterogeneity study is urgently needed.

Herein, we proposed a label free cell identification technology utilizing ratiometric mass spectrometric strategy. Although the amounts of molecules have wide dynamic range and each shows different desorption and ionization ability in the MALDI MS, several pairs of peptides and proteins with similar molecular weight can be regarded as internal standards for each other, especially for those sharing similar structure. In the present study, hepatocellular carcinoma (HCC) cell lines were used as model cell lines. We found that the relative intensity of peak pairs at *m/z* = 4936 vs 4962 and *m/z* = 9952 vs *m/z* = 10088 detected in the cell lines are highly conserved. When different species of cells were mixed or co-cultured, the ratiometric peak information can be utilized as a “cell-ID” tag for quantitative analysis. Based on this finding, different types of HCC cells can be rapidly identified and quantified according to the ratio values of these peak pairs in mass spectra. Coupled with MALDI imaging technology, distribution and proportion of cell types in tumor tissue can be estimated. The established technology is simple, quick, and of high throughput. It may become a powerful tool in tumor heterogeneity study and cell identification in complex biological sample.

## Results and Discussion

### Identification of HCC cells by MALDI-TOF MS

**Figure 1:**
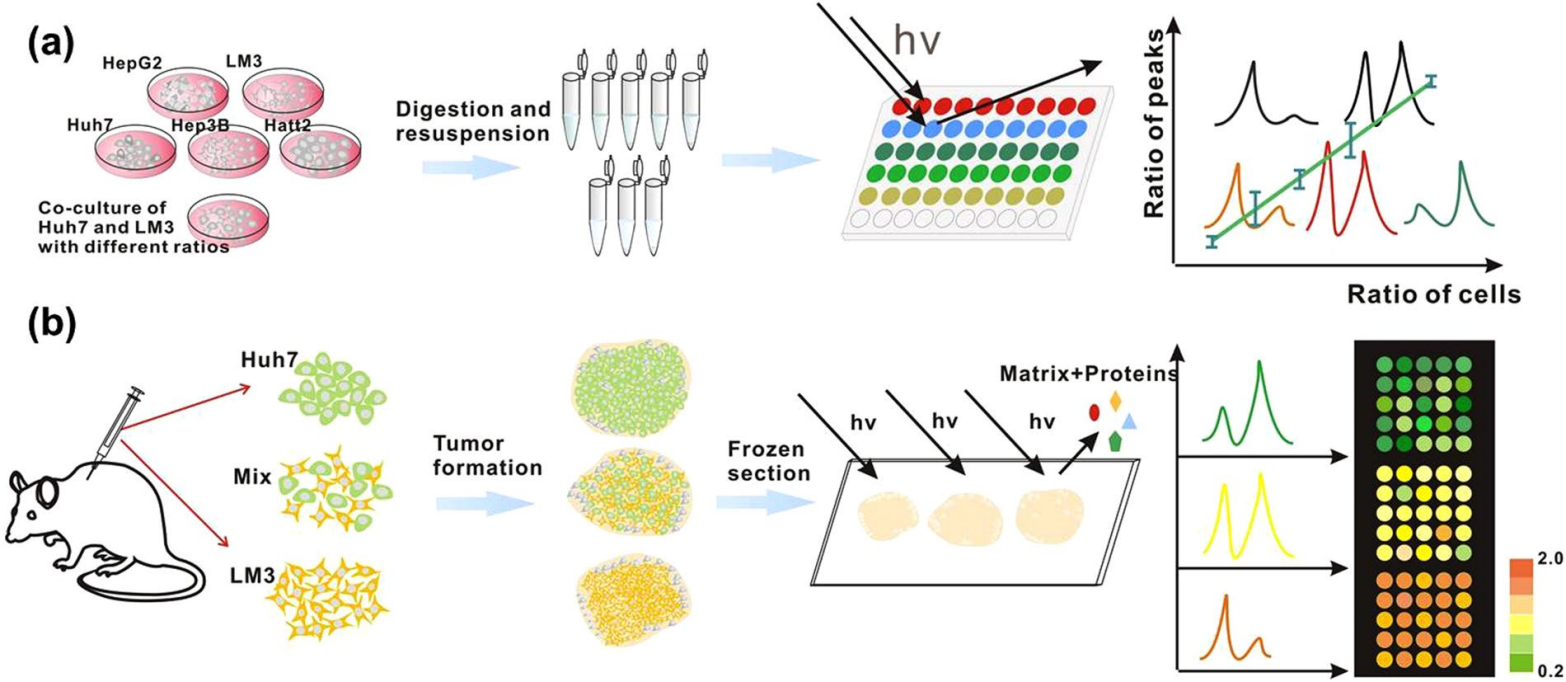
(**a**) Whole-cell MS detection strategy and (**b**) On-tissue MS profiling for cell identification.

**Figure 2:**
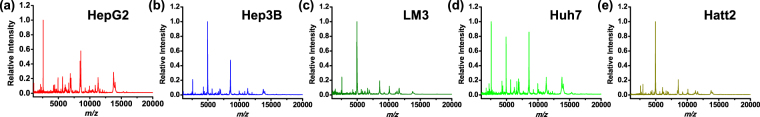
Typical normalized MALDI mass spectra of five types of hepatocellular cancer cells (HCC cells). (**a**) HepG2, (**b**) Hep3B, (**c**) LM3, (**d**) Huh7, (**e**) Hatt2. Spectra have been normalized within [0, 1] by dividing the max peak intensity in each spectrum.

To prove the applicability of cell identification by MALDI-TOF MS, five types of HCC cells named as HepG2, Hep3B, Huh7, LM3 and Hatt2 cells were chosen as the models. In the detection of HCC cells by MALDI-TOF MS, a whole-cell detection strategy was used, as illustrated in Fig. 1a. All gathered cell suspensions were immediately stored in −80 °C refrigerator without extra chemical or biochemical lysis process. To avoid interference with the MS detection, protease inhibitors for elimination of protein degradation were not added. For the matrix application, CHCA was chosen as a better peptide/protein extractor in the low molecules weight regions than SA and DHB (Figure S1). Other measurement conditions were optimized and were consistent in all experiments (See Methods). Using the standard operation and treatment protocol, the mass spectra of cells kept almost stability in different independent tests (Figure S2). As shown in Fig. 2, each type of HCC cells shows different mass fingerprint. To avoid the effect of cell variation in the propagation, mass spectra of each type cell were acquired from 15 cell samples, which were gathered from five continuous generations with three replicates in each generation (Figure S3). The results indicated that the mass profile among different generations was quite consistent for each type of cell sample. Take into consideration of the culture environment effect on the protein expression of cells, each type of cells was cultured in three different common-used media, DMEM, MEM and RPMI 1640, respectively. Each type of cells displayed similar mass fingerprints in different culture media (Figure S4), while distinct mass patterns were observed among different types of cells. The results indicated that the cell mass fingerprints were highly related to the cell type, not the culture medium.

**Figure 3:**
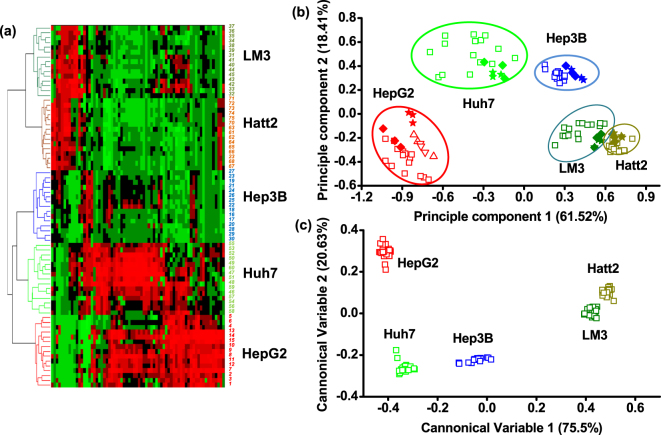
(**a**) Hierarchical clustering (HCL) analysis of five HCCs cultured in DMEM medium. Average Pearson correlation method was used in the HCL analysis. For each type of cells, spectra were acquired from 15 cell samples (No. 1~15 HepG2, No. 16~30 Hep3B, No. 31–45 LM3, No. 46~60 Huh7, No. 61~75 Hatt2), which were gathered from five continuous generations. In other figures, all representative mass spectra acquired in DMEM culture medium were referred to No. 11 for HepG2, No. 18 for Hep3B, No. 41 for LM3, No. 46 for Huh7 and No. 68 for Hatt2. (**b**) Principle component analysis (PCA) of five types of HCC cells. Two principle components percentage were 61.52% and 18.41%, respectively. Open squares (◽) were referred to the 75 samples used in the HCL analysis; open triangles (▵/▿) in HepG2 circle meant another two independent tests; solid rhombuses (♦) were referred to the cells cultured in MEM while solid stars (★) were referred to the cells cultured in RPMI 1640. HCL and PCA were all based on the 70 feature peaks selected by ClinproTools (p < 0.01). (**c**) Linear discrimination analysis (LDA) of five types of HCC cells based on the 11 predominated principle components (contribution > 0.1%) of the PCA result. Cells cultured in different media were also included in the PCA-LDA model construction.

To select feature peaks for cell discrimination, proper data treatment and statistical analysis were performed. First, five groups of cell mass spectra were pretreated with smoothing and baseline subtraction in the FlexAnalysis software. All the pretreated mass spectra were normalized before statistical analysis. Then, the processed spectra were exported into ClinproTools software, which can provide a list of peaks sorting by statistical significance to differentiate between each two classes using default settings. When the *P* value was set below 0.01, 70 feature peaks (Table S1) were selected after validation with the real spectra in FlexAnalysis. Using these feature peaks, hierarchical clustering (HCL) and principle component analysis (PCA) were completed in MATLAB software. As shown in the cluster tree and PCA graph, each type of HCC cells can be classified into their respective groups (Fig. 3a,b), further confirming that the replicated tests in different times and the culture media would not influence the PCA results (Fig. 3b). When linear discriminated analysis (LDA) was conducted based on the 11 predominated principle components (contribution >0.1%) of the PCA, five types of cells can be accurately discriminated from each other (Fig. 3c). The PCA-LDA model can be applied for cell type identification, since the testing prediction accuracy was 100% when the training set and testing set occupied 85% and 15% of the data set, respectively. The results inferred that the panel of mass spectral peaks can be regarded as cell-ID barcode for cell discrimination and identification due to its unique molecular pattern. In the future study, more mass fingerprints of various tumor cells need to be acquired for database construction.

### Quantitative analysis of HCC cells using intracellular double biomarkers

**Figure 4:**
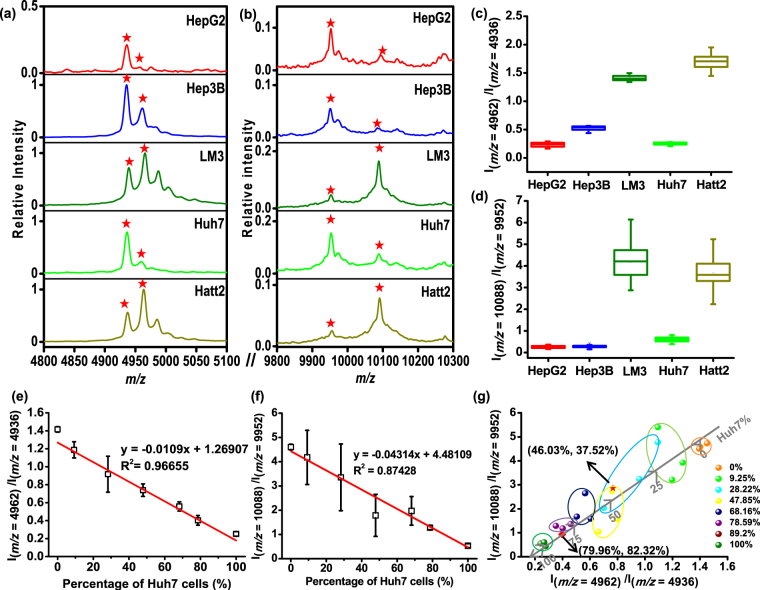
(**a**,**b**) Typical magnified MALDI MS spectra of five types of HCC cells at (**a**) *m/z* = 4962 vs 4936 and (**b**) *m/z* = 10088 vs 9952. (**c**,**d**) Peak intensity ratio of (**c**) *m/z* = 4962 to 4936 and (**d**) *m/z* = 10088 to 9952 detected in five types of HCC cells. The ratio of peak pairs for each type of cell is an averaged value calculated from 15 replicates. The RSD of Ratio 1 (I_(m/z = 4962)_/I_(m/z = 4936)_) for five types of HCCs were 16.4%, 7.9%, 3.4%, 9.9%, 7.5% and the RSD of Ratio 2 (I_(m/z = 10088)_/I_(m/z = 9952)_) for five types of HCCs were 24.2%, 23.4%, 20.3%, 20.8%, 19.9%, respectively. (**e**,**f**) The relationship between peak intensity ratio of (**e**) *m/z* = 4962 to *m/z* = 4936, (**f**) *m/z* = 10088 to *m/z* = 9952 and percentage of Huh7 cell in the mixture composed of Huh7 and LM3 cells. (**g**) 2D scatter graph of two pairs of dual biomarker ratio. The value shown in the brackets are the calculated Huh7% according to the fitting curve in (**f**) and (**g**), respectively. The red square and star in (**g**) are referred to the co-cultured samples with an initial ratios of Huh7/LM3 = 1:1 and 3:1, respectively.

Different types of cells show specific mass spectral profiles, whose featured molecular panel can be regarded as ID tag for cell identification. However, the quantification ability of MALDI technology is short of expectation, due to the non-uniformity of matrix crystallization and other inherent factors^41^. If different types of cancer cells coexist, the relative quantitation is not easy. As shown in Figure S5, though cell mixture can be discriminated from pure cells through PCA, the proportional information cannot be obtained. The common method to realize cell quantitation is to use isotope labeling techniques. Nevertheless, all those label-based methods are time-consuming and inconvenience. In the current work, we found that several pairs of peptides or proteins with similar molecular weight exist in the HCC cells. Their relative abundance was highly conserved and therefore can be regarded as internal standards for each other. It was supposed that these pairs of peaks may possess similar desorption and ionization efficiency and the relative intensity would not be largely influenced by matrix suppression effect^42^. The finding of the peak pairs for internal standard is based on 70 feature peaks selected for cell identification as mentioned above. After calculating the ratio value of these peak pairs whose relative difference is within 2.5% of their average molecular weight, 11 pairs of dual-peaks were selected out, showing relative standard deviation (RSD) <25% in each type of cells (Figure S6). However, among these pairs, only the dual-peaks at *m/z* = 4894 vs 4962, *m/z* = 4936 vs 4962, *m/z* = 6136 vs 6174 and *m/z* = 9952 vs 10088 showed significant differences between at least four types of HCC cells after Student’s t test (Table S2). Finally, the two peak pairs at *m/z* = 4936 vs 4962 and *m/z* = 9952 vs 10088 were scrutinized due to the quite low intensity of the peak at *m/z* = 4894, 6136 and 6174 in the mass spectra. In addition, the ratio values of these selected two pairs were quite stable in different generations (Fig. 4c,d), as well as in different culture circumstance (e.g. different culture media) (Figure S7). Peaks at *m/z* = 4936 and *m/z* = 4962 were the feature peaks account for the largest proportion of principle component 1 and 2 in PCA, respectively (Table S3). Accordingly, based on these two peak pairs, a two-dimensional ratiometric mass spectral approach was employed for cel quantitation.

As a proof-of-concept experiment, Huh7 and LM3 cells were used as quantitative models by mixing their suspension with different percentages. The mass fingerprint of cell mixture looked like an overlay of the spectra belonging to Huh7 and LM3 (Figure S8). Importantly, the ratios of the two peak pairs exhibited linear relationship with the proportion of Huh7 cells (Fig. 4e,f). Compared with the pair at *m/z* = 9952 vs *m/z* = 10088, the ratio value of *m/z* = 4936 vs *m/z* = 4962 was much more stable. For all mixed samples, the relative standard deviations of this ratio value (I_(*m/z *= 4962)_/I_(*m/z *= 4936)_) for three parallel measurements were around 2.19% and 21.8% with correlation coefficient (R^2^) = 0.96655. Though the pair at *m/z* = 9952 and *m/z* = 10088 exhibited more uncertainty, its ratiometric data can provide supplementary information for cell quantitation. As shown in Fig. 4g, the two-dimensional ratiometric data can further ensure the accuracy of quantitative analysis at the cell level. To validate the ratiometric method for cells quantification, a blind test was performed. In order to simulate the cell heterogeneous condition, Huh7 and LM3 cells were co-cultured with known initial proportions at the passage process. For the sample 1 and sample 2, the initial ratios of Huh7/LM3 were 1:1 and 3:1, respectively. After two days’ proliferation, both types of cells were propagated to the logarithmic growth phase. The propagated cells were gathered and subjected to MALDI MS detection and data acquisition. As the relative growth rates of these two types of cells were unknown, the final ratio of cell numbers kept anonymous. By referring to the linear curve shown on Fig. 4e,f, the percentage of each type of cells can be calculated accordingly. To our expectations, the averaged percentage of Huh7 in the sample 1 calculated from the two separate fitting curves were 46.03% and 37.52%, respectively, while the values in sample 2 were 79.96% and 82.31%. It can be found that the calculated data in each sample from two fitting curves maintained inner consistent and all the ratio data can fit well with the curve in 2-D graph shown in Fig. 4g. The position of scattered dots in the 2-D curve was highly correlated with the initial composition of cell mixture.

**Figure 5:**
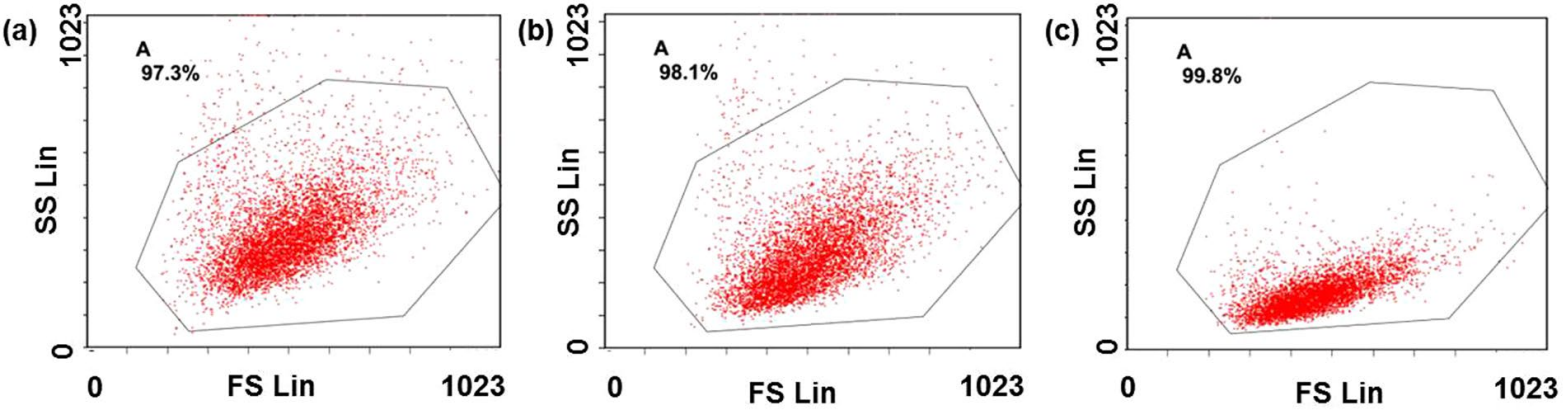
Flow cytometry results of (**a**) Huh7 cells, (**b**) cell mixture with a ratio of Huh7/LM3 = 1:1, (**c**) LM3. X axis is forward scatter (FS Lin) which is related to the size of cells while Y axis is the side scatter (SS Lin) which is related to the granularity of cells.

Though flow cytometry is also effective for cell counting and relative quantification, only those cells with large difference in size and shape (e.g. blood cells and cancer cells) can be easily sorted. For the tumor cells sharing similar physical properties, fluorescence labeling is indispensable. However, cells with unknown surface acceptors cannot be effectively discriminated and quantified with the labelling methods. As shown in Fig. 5, Huh7 and LM3 cannot be clearly discriminated using flow cytometry just relying on light scattering signals. In contrast, the ratiometric mass strategy in this work is label free and high throughput. Different HCC cells can be identified and quantified using the specific intracellular dual-biomarkers. Though HepG2 vs Huh7, HepG2 vs Hep3B show no significant difference just using one pair peaks, they can be still semi-quantitatively discriminated by another ratio value of the selected two pairs (Figure S9). In addition, the “ratio of these two ratio values” can also be defined as the third quantification standard since most cells demonstrated significant difference of this ratio except LM3 vs Huh7 and Huh7 vs Hatt2 (Figure S10). This supplementary information may ensure the quantification accuracy for those cells sharing similar ratio value of I_(*m/z*=4962)_/I_(*m/z*=4936)_ or I_(*m/z*=10088)_/I_(*m/z*=9952)_ on the premise of qualitative identification by mass fingerprint of different cells.

### Application of intracellular dual biomarkers in the identification of tumors

To test whether the ratiometric approach can be further applied in the identification of cells in a real tumor sample, Huh7 and LM3 model cells were subcutaneously co-injected into the nude mice, simulating the heterogeneity in the tumor tissue (Fig. 1b). In the inoculation procedure, as the proportion of cells in different region within a tumor cannot be artificially controlled, the mixed cells (Huh7 and LM3) with initial proportion of 1:0, 3:1 and 0:1 were subcutaneously injected into different nude mice, respectively. After the tumors with needed size (~1 cm^3^) were formed, mice were sacrificed for tumor gathering and tissue frozen section. On each section of tissues, 25 spots composed of a 5 × 5 array were applied for MALDI MS detection after the tissue section was homogeneously sprayed with CHCA matrix.

**Figure 6:**
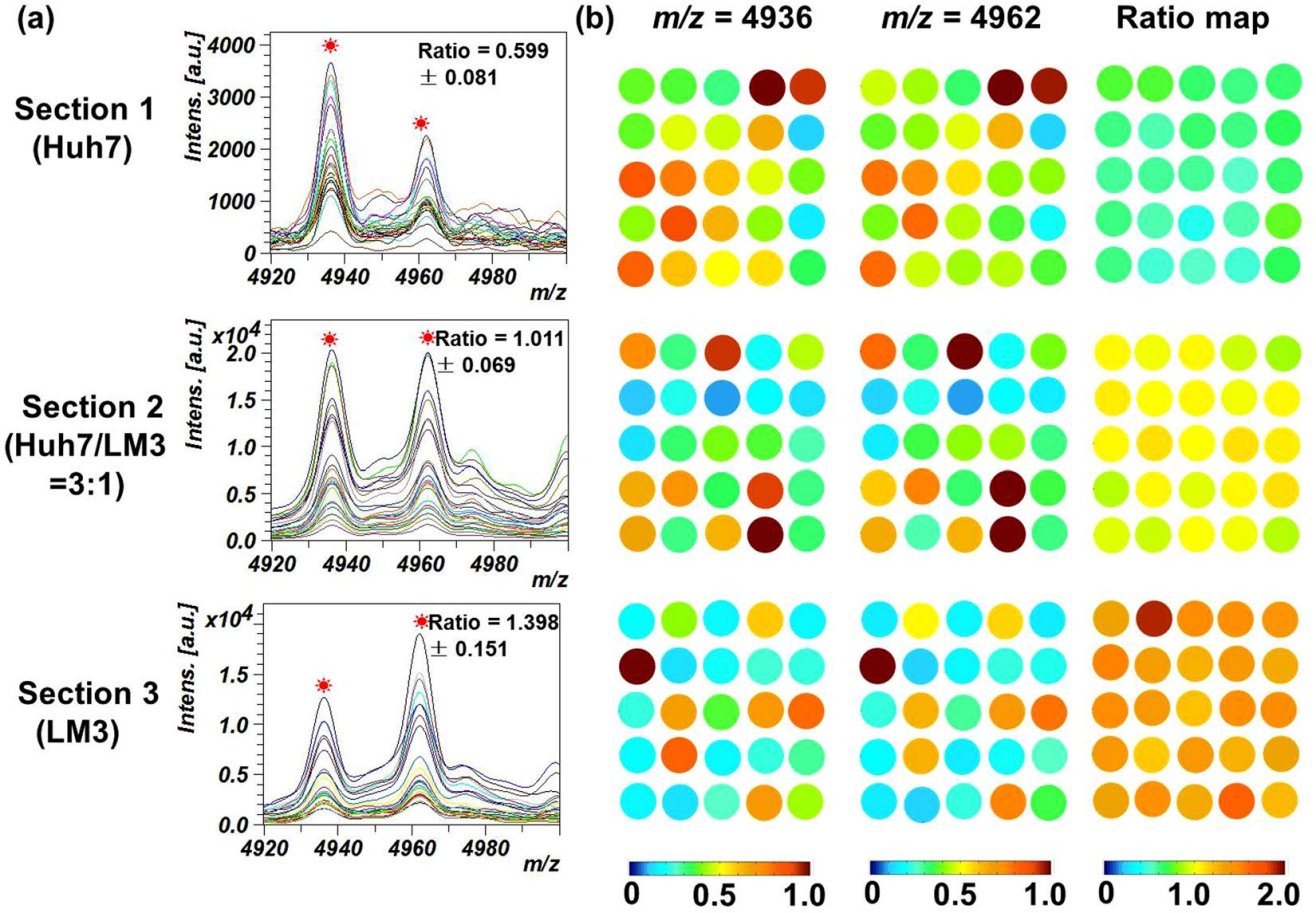
(**a**) MALDI mass spectra of the tissues originated from pure Huh7 cells, pure LM3 cells and mixed cells with a ratio of Huh7/LM3 = 3:1 at the *m/z* = 4936 and *m/z* = 4962. (**b**) Heatmaps of different tumor tissue sample derived from the peak intensity at *m/z* = 4936, *m/z* = 4962 and the ratios of these two peaks. On each tissue section, a 5 × 5 spots array with the diameter of 1000 μm was applied for mass spectra acquirement. Heatmaps of these two distinctive signals were normalized within [0, 1] by dividing the max value while the ratio value range was set within [0.2, 2.0].

As shown in Figure S11, the pattern of MS spectral profile acquired from the tissues seem different with that obtained from cells (Fig. 2, Figure S3), which might be caused by different methods for sample preparation, matrix coating and extracting processes. However, when the peak pair at *m/z* = 4936 vs 4962 were used, we found that their ratio was almost constant among the same type of cell samples. When the normalized intensity of these two peaks as well as their ratio values were extracted and exported into the MATLAB software, a digital color map can be formed (Fig. 6). If we only use the intensity of one peak for MS imaging, the heatmaps of the 5 × 5 array display large fluctuations, owing to the non-uniformity of matrix crystallization or inherent shortage of MALDI technology. The uncertainty of absolute peak intensity may give false results when cell identification in different tissue region is required. Nevertheless, the heatmaps derived from the peak intensity ratio of the dual biomarkers can visualize the percentage of two model cells with high fidelity owing to the high conservation of the ratio value. For the tissue grown from pure Huh7 cells, all detection spots in the 5 × 5 array displayed blue-greenish color because the pure Huh7 cell has a lower ratio in the intensity of the dual peaks. In contrast, the heatmap of tissue grown from LM3 tended to be orange, which was determined by the characteristics peak ratio of LM3 cell. When the initial proportion of mixed cells (Huh7/LM3) was 3:1, the color of the tissue tended to be intermediate between the samples composed by pure Huh7 and LM3 cells. Though the peaks at *m/z* = 4936 and *m/z* = 4962 also appeared in the surrounding muscle tissue as well as the normal liver tissue (Figure S11d,e), the ratio values of these two kinds of tissues exceeded 4, which showed largely significant difference from the HCC tumor models (Figure S11f). Both cell-based and tissue-based experiments confirm that ratiometric mass spectral approach is a feasible tool for cell quantitation and identification in complex biological samples.

### The identification of the dual biomarkers

Previous study on cell lysis^12^ and tissue imaging (http://129.187.44.58:7171/MALDI/protein/) have proposed that the peptides with *m/z* = 4962 and *m/z* = 4936 belong to thymosin β4 and thymosin β10. Both of them are a 43-amino acid polypeptide sharing similar primary structure, while several different amino acids account for the subtle difference of molecular weight^43^. In the present work, the existence of the two peptides in the HCC cell lines samples were also verified by LC-MS/MS identification (Figure S12). Up to now, several works in the fields of biology and medicine have reported the function of thymosin molecules, which are considered to be widely distributed in many tissues and play a vital role in cell migration, angiogenesis and cancer malignancy^44–47^. However, the exact role of the thymosin family in cancer is still unclear due to the dispute point of views in different literatures^47–49^. Up to now, the biochemical meanings of the relative content of these two polypeptides haven’t been noticed. The stable ratio of this thymosin pair corresponding to each kind of HCC cells is an important finding of this work. In addition, normal tissue in the model mouse, such as muscle and liver, displayed a relative low intensity of *m/z* = 4936 compared to the peak at *m/z* = 4962, which showed significant difference from the HCC tumor models. Based on this finding, it can be expected that the ratio value change of thymosin pair might become an indicator related with cancer cell proliferation, migration and activation. As to the other pair at *m/z* = 9952 vs 10088, the identification and their biochemical function need to be further investigated. Future work on this biomarker pair will be conducted.

## Conclusion

In summary, a convenient and high-throughput method for cell identification and quantitation has been established by ratiometric MALDI MS. Using selected feature peaks obtained in molecular profile, different types of HCC cell lines can be easily differentiated with cluster and principle component analysis. Furthermore, two peak pairs whose ratio of intensity is highly related with the type of HCC cells and their proportions were found, that will greatly simplify the cell identification and quantitation processes. LC-MS/MS identified that one peak pair *m/z* = 4962 and *m/z* = 4936 belong to thymosin β4 and thymosin β10. The feasibility of the ratiometric mass spectral approach has been validated either in co-cultured cell samples and real tumor samples, demonstrating its potential ability for intra-tumor heterogeneity study. What’s more, the ratiometric approach may result in a new concept for future clinical diagnosis. Various evidences have suggested that mono-biomarker, such as α-foetoprotein (AFP), human epidermal growth factor receptor II (HER2), prostate specific antigen (PSA), carcinoembryonic antigen (CEA), etc. often tell false positive or false negative results when the clinical criterion was set at an absolute level^50–53^. The ratiometric approach based on intracellular dual-biomarker detection may provide us a more reliable way to monitor and classify disease.

## Methods

### Materials and reagents

Three common used culture media, DMEM, MEM and RPMI 1640 and trypsin−EDTA were purchased from Gibco Corp. (Grand Island, NY, USA). Fetal bovine serum (FBS) was purchased from Biological Industries Corp. (Israel). HPLC grade ethanol (EtOH), acetonitrile (ACN) and trifluoroacetic acid (TFA) were purchased from Sigma-Aldrich Corp. (Shanghai, China). Sequencing grade modified trypsin was purchased from Promega Corp. (Beijing, China). Matrigel® basement membrane matrix (No. 356234) was purchased from Becton Dickinson Corp. (USA). The MALDI matrix, α-cyano-4-hydroxycinnamicacid (CHCA), sinapic acid (SA) and 2, 5-dihydroxybenzoic acid (DHB) and Indium Teen Oxide (ITO)-coated glass slides as well the protein/peptide calibration standards (700–25000 Da) were the products from Bruker Daltonics Corp. (Germany). H&E staining kit was purchased from Beyotime Biotechnology Corp (Shanghai, China). HCC cell lines, HepG2 and Hep3B were obtained from American Tissue Culture Collection. Huh7, LM3 and Hatt2 cells were obtained from the Cell Bank of the Chinese Academy of Science (Shanghai, China) and male nude mice were purchased from Slack Corp. (Shanghai, China). All other reagents used in this experiment were of analytical reagent grade and used without further purification.

### Cell culture and sample preparation

Five types of HCC cells named as HepG2, Hep3B, Huh7, LM3 and Hatt2 cells were cultured in DMEM medium complemented with 10% FBS in the cell incubator with 5% CO_2_ at 37 °C. The cells were reseeded every 2 or 3 days. After reaching 80–90% confluence, the cells were treated with trypsin-EDTA. Subsequently, the cells were centrifuged and the supernatant was discarded. The cell pellet was subsequently washed twice with PBS solution before re-suspended in PBS to obtain a cell suspension with the concentration at 1 × 10^7^ cells/mL. For each type of HCC cells, cell suspensions from five continuous generations were gathered with three replicates. To verify the stability of cell mass fingerprints in different culture environment, these HCC cells cultured in MEM and RPMI 1640 media were also gathered for MS detection with the same protocol. To get mixed suspensions of different cells, cell suspensions with the same concentration were mixed by several certain ratios. In order to verify the feasibility of ratiometric information for quantitative identification in a more complex model, Huh7 and LM3 cells with different ratios were co-cultured for 2 days before trypsinization and resuspension in PBS. Finally, all needed cell suspensions were stored in −80 °C refrigerator before MALDI MS analysis.

### Tissue preparation

Huh7 and LM3 cell suspensions were centrifuged and then mixed with different proportions and re-suspended in DMEM complemented with 50% Matrigel^®^ to obtain a solution containing 10^7^ cells/mL. Cells were injected subcutaneously in the dorsal region of three nude mice. The cell number for each tumor was controlled at ~2 × 10^6^. Pure Huh7 and LM3 cells were injected into the mice No. 1 and No. 3, while the mixed cells with a ratio of Huh7/LM3 = 3:1 were injected into the mice No. 2. Around 30 days after inoculation of cells, the dimension of tumors was approximately 1 cm^3^ and the mice were sacrificed for tumor removal. At the same time, the surrounding muscle tissue and the liver tissue from the nude mouse were also acquired. To minimize the protein degradation and morphological change, all collected tumor tissue samples were quickly frozen in liquid nitrogen and stored at −80 °C before sectioning. Tumor tissues were sliced into approximately 12 μm thickness at −20 °C with a Leica CM30503 cryostat (Leica Microsystems, Germany). Then the thawed tissue slice was mounted onto an ITO-coated glass slide. The slides with the tissue sections were rinsed with 70% ethanol and 96% ethanol for 60 s each. Following washing with ethanol, the samples were dried in vacuum for 30 min and stored at −80 °C until matrix coating. All animal studies were performed in accordance with the guidelines of the Laboratory Animal Center of Zhejiang University (License Number: SYXK 2012-0178). And all animal experimental protocols were approved by the the Laboratory Animal Center of Zhejiang University.

### MALDI MS identification and tissue imaging profiling

Prior to MALDI measurement, cell samples were thawed at 4 °C first and rotated to be homogeneous on a vortex. Then a 2 μL droplet of cell suspensions was deposited onto the MALDI target. After drying of the cell suspension liquid, 2 μL saturated CHCA dissolved in a mixture of water, ACN and TFA (50:50:0.1, v/v/v) were dropped onto the analyte and waited to be dry at RT (300 K). MALDI-TOF mass spectra were obtained on the UltrafleXtreme MALDI-TOF/TOF instrument (Bruker Daltonics Corp.) equipped with a 355 nm Nd:YAG laser beam (pulse energy < 500 μJ with a pulse width = 3 ns). The relative laser pulse energy was set at 87.9% of the maximum energy. The ions that resulted from a 250 ns pulse ion delayed extraction were subjected to an acceleration voltage of 25 kV (ion source 1) and 23.65 kV (ion source 2) and analyzed in the positive linear mode. The generated spectra were obtained by averaging data from 500 individual laser shots. For each spot, five replicated mass spectra were added to eliminate the variation of single mass spectrum.

As for the MS analysis of tissue sections, 7 mg/mL CHCA matrix was sprayed onto the samples by the ImagePrep instrument (Bruker Daltonics Corp.). On each tissue section, a 5 × 5 spots array with the diameter of 1000 μm was applied for mass spectra acquirement with the same method used in the cell suspension detection in AutoXecute mode, representing the overall information of the tissue section. After MS detection, the tissue sections were washed with EtOH to get rid of the matrix crystals. After that, the tissue sections were stained with Hematoxylin and Eosin for cell observation in tissue sections.

### Statistical analysis

The acquired raw mass spectra were first pretreated with baseline subtraction and smoothing once each in succession in the FlexAnalysis 3.4 software (Bruker Daltonics Corp.). Then the mass spectra were exported as ASCII data. To obtain the standard mass spectra, these data were normalized by dividing the max value in each spectrum. The selection of feature peaks was completed by ClinproTools software (Bruker Daltonics Corp.) following importing mass spectra, which can provide a list of peaks sorting by Student’s t testing to differentiate between each two classes when the *P* value was set below 0.01. Using the normalized data of these feature peaks, hierarchical clustering (HCL) and principle component analysis (PCA) were completed in MATLAB software. An average Pearson correlation method was used in the HCL analysis. After PCA, the linear discriminated analysis (LDA) was conducted in MATLAB based on the 11 predominated principle components (contribution > 0.1%). In the PCA-LDA model, the training set occupied 85% of the data set, while the other 15% data were used as the testing set for evaluation of prediction accuracy.

### Flow cytometry detection of cells

0.5 mL pure Huh7 cells, LM3 cells and cell mixture of Huh7/LM3 = 1:1 with a concentration at 10^6^ cells/mL suspended in PBS were applied for flow cytometry detection on the Cytomic FC 500MCL flow cytometer (Beckman Coulter Corp.). Forward scatter and side scatter without fluorescence channels were used to sort cells.

### LC-MS/MS identification of selected biomarkers

To acquire the amino acid sequencing information of the detected peptides and proteins, LC-MS/MS identification was applied for the trypsin-treated LM3 cell samples. Peptides and proteins of the cell samples with a concentration of 10^7^ cells/mL were firstly extracted by 50 mM ammonium bicarbonate, along with three repetitive freeze-thawing processes and 5 min sonication on ice. After extraction, the mixture was centrifuged at 15,000 rpm/min for 5 min and the supernatant was collected. 4 μL of 0.1 mg/mL trypsin in 50 mM acetic acid was added into the 200 μL lysis supernatant and the sample was incubated for about 16 hours at 37 °C. Before LC-MS/MS detection by the LTQ Orbitrap Elite MS instrument (Thermal Fisher Scientific Corp.), the sample was firstly desalted. Then the sample was injected onto the trap column with a flow rate of 10 μL/min and subsequently eluted with a five-step linear gradient (A: ddH_2_O with 0.1% formic acid, B: ACN with 0.1% formic acid): 0–10 min, 3–8% B; 10–120 min, 8–20% B; 120–137 min, 20–30% B; 137–143 min, 30–90% B; 143–150 min, 90% B. Data collection by Fourier transform ion cyclotron resonance mass analyzer (FTMS) was then proceeded to isolate the top 20 ions for MS/MS by CID. Proteome Discover software (Thermal Fisher Scientific Corp.) was applied for sequencing matching of detected MS/MS spectra.

## Electronic supplementary material

Supplementary Information
