# Supplementary material for: Ratiometric Mass Spectrometry for Cell Identification and Quantitation Using Intracellular “Dual-Biomarkers”
Source: Sci Rep. 2017 Dec 12;7:17432. doi: 10.1038/s41598-017-17812-1 (PMC5727126; doi:10.1038/s41598-017-17812-1)
Supplement: Supplementary file 1 — Supplementary Information [file 41598_2017_17812_MOESM1_ESM.pdf]

Supporting Information

Ratiometric Mass Spectrometry for Cell Identification and Quantitation Using Intracellular "Dual-Biomarkers"

Xiaoming Chen[1], Fangjie Wo[1], Jiang Chen[2], Jie Tan[1], Tao Wang[1], Xiao Liang[2]*, Jianmin Wu[1]*

[1]*Institute of Analytical Chemistry, Department of Chemistry, Zhejiang University, Hangzhou, 310058, China*
[2]*Department of General Surgery, Sir Run Run Shaw Hospital, School of Medicine, Zhejiang University, Hangzhou, 310016, China*

***Corresponding author**: Jianmin Wu, e-mail: wjm-st1@zju.edu.cn

Table of Contents

Supplemental Figure S1 Normalized MALDI mass spectra of HepG2 cells with different organic matrix

Supplemental Figure S2 Typical Normalized MALDI mass spectra of HepG2 cells acquired in different independent tests

Supplemental Figure S3 Normalized MALDI mass spectra of five types of hepatocellular cancer cells (HCC cells)

Supplemental Figure S4 Normalized MALDI mass spectra of five types HCC cells cultured in DMEM, MEM and RPMI 1640 media.

Supplemental Figure S5 Principle component analysis (PCA) of cell mixture and pure cells

Supplemental Figure S6 Magnified mass spectra of five type HCC cells at 11 pairs of dual-peaks whose RSD < 25% in each type of cells

Supplemental Figure S7 Magnifed mass spectra at *m/z* = 4962 vs 4936 and *m/z* = 10088 vs 9952 of five type HCC cells cultured in three different media

Supplemental Figure S8 Typical normalized MALDI mass spectra of LM3, Huh7, mixture of Huh7 and LM3 with ratio = 1:1 cultured in DMEM

Supplemental Figure S9 The relationship between peak intensity ratio and cell mixture composition of Huh7 and HepG2 cells, Hep3B and HepG2

Supplemental Figure S10 Ratio of two ratio values (Ratio 1 = $I_{(m/z = 4962)}$/ $I_{(m/z = 4936)}$, Ratio 2 = $I_{(m/z = 10088)}$/ $I_{(m/z = 9952)}$) in five types of HCC cells and the *P* values between different types of cells

Supplemental Figure S11 MALDI mass spectra of the tissues originated from pure Huh7 cells, LM3 cells, mixed cells with a ratio of Huh7/LM3 = 3:1, nude mice muscle and liver tissue

Supplemental Figure S12 LC/MS MS identification of thymosin β 4 and thymosin β 10

Supplemental Table S1 Normalized intensity of 70 feature peaks from the mass spectra of five types of HCC cells selected by ClinproTools software

Supplemental Table S2 *P* value calculated with Student’s test of the 11 dual-peaks between each two classes of five types of HCC cells

Supplemental Table S3 Feature peaks information ranking first in the principle component analysis

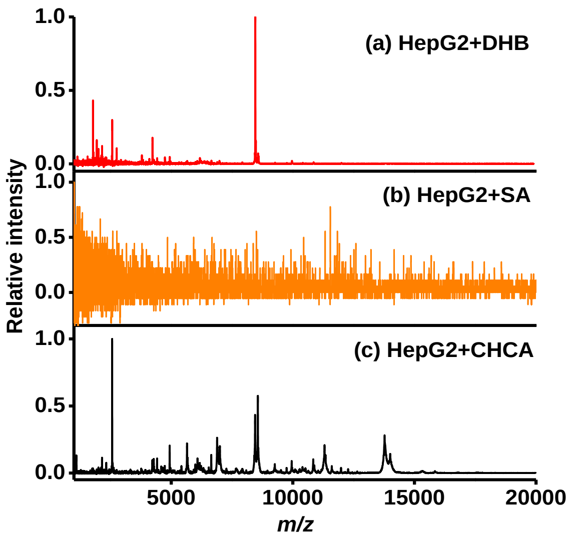

Figure S1. Normalized MALDI mass spectra of HepG2 cells with different organic matrix. (a) DHB (2, 5-Dihydroxybenzoic acid), (b) SA (Sinapic acid), (c) CHCA (α-Cyano-4-hydroxycinnamic acid). All matrix were prepared as saturated solutions whose solvent was a mixture of water, ACN and TFA (50:50:0.1, v/v/v). Spectra have been normalized by dividing the max peak value in each spectum.

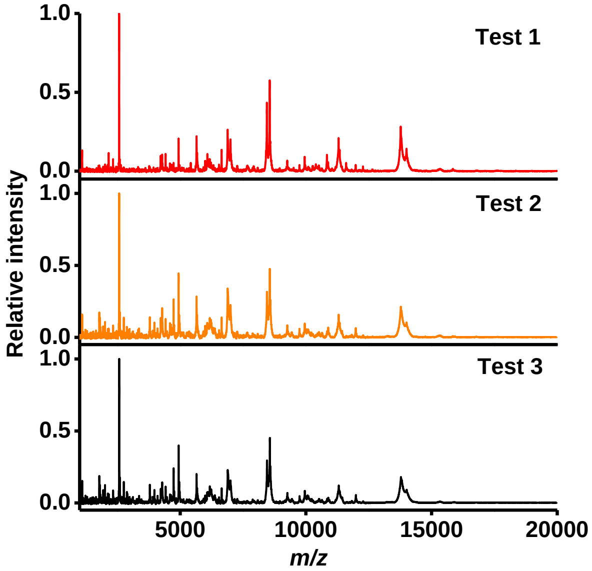

Figure S2. Typical normalized MALDI mass spectra of HepG2 cells acquired in different independent tests. The three tests were completed in three different days and the samples were obtained in the same culture generation. Spectra have been normalized by dividing the max peak value in each spectrum.

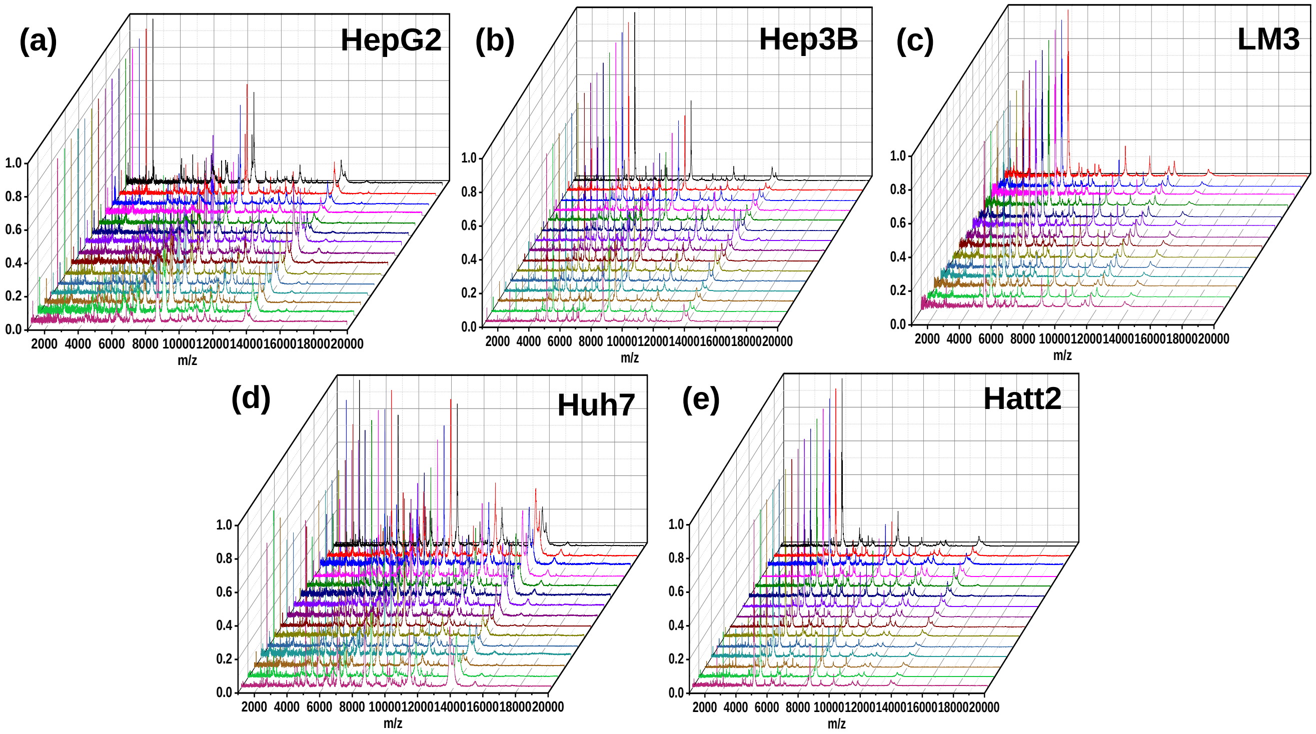

Figure S3. Normalized MALDI mass spectra of five types of hepatocellular cancer cells (HCC cells). (a) HepG2, (b) Hep3B, (c) LM3, (d) Huh7, (e) Hatt2. For each type of cells, spectra were acquired from 15 cell samples, which were gathered from five continuous generations with three replicates in each generation. Spectra have been normalized by dividing the max peak value in each spectum.

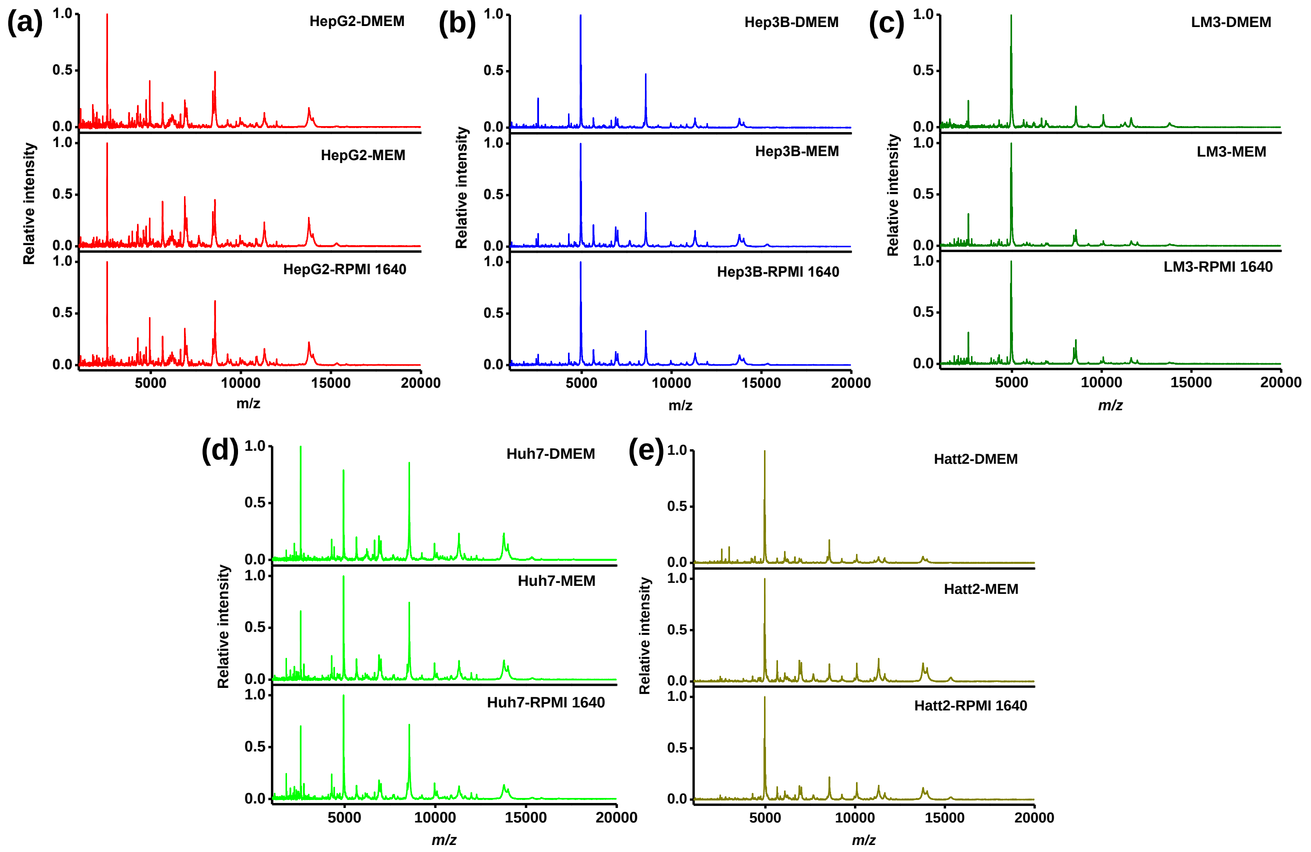

Figure S4. Normalized MALDI mass spectra of five types HCC cells cultured in DMEM, MEM and RPMI 1640 media. (a) HepG2, (b) Hep3B, (c) LM3, (d) Huh7, (e) Hatt2. Mass spectra of cells cultured in DMEM are the same with those in Figure 2.

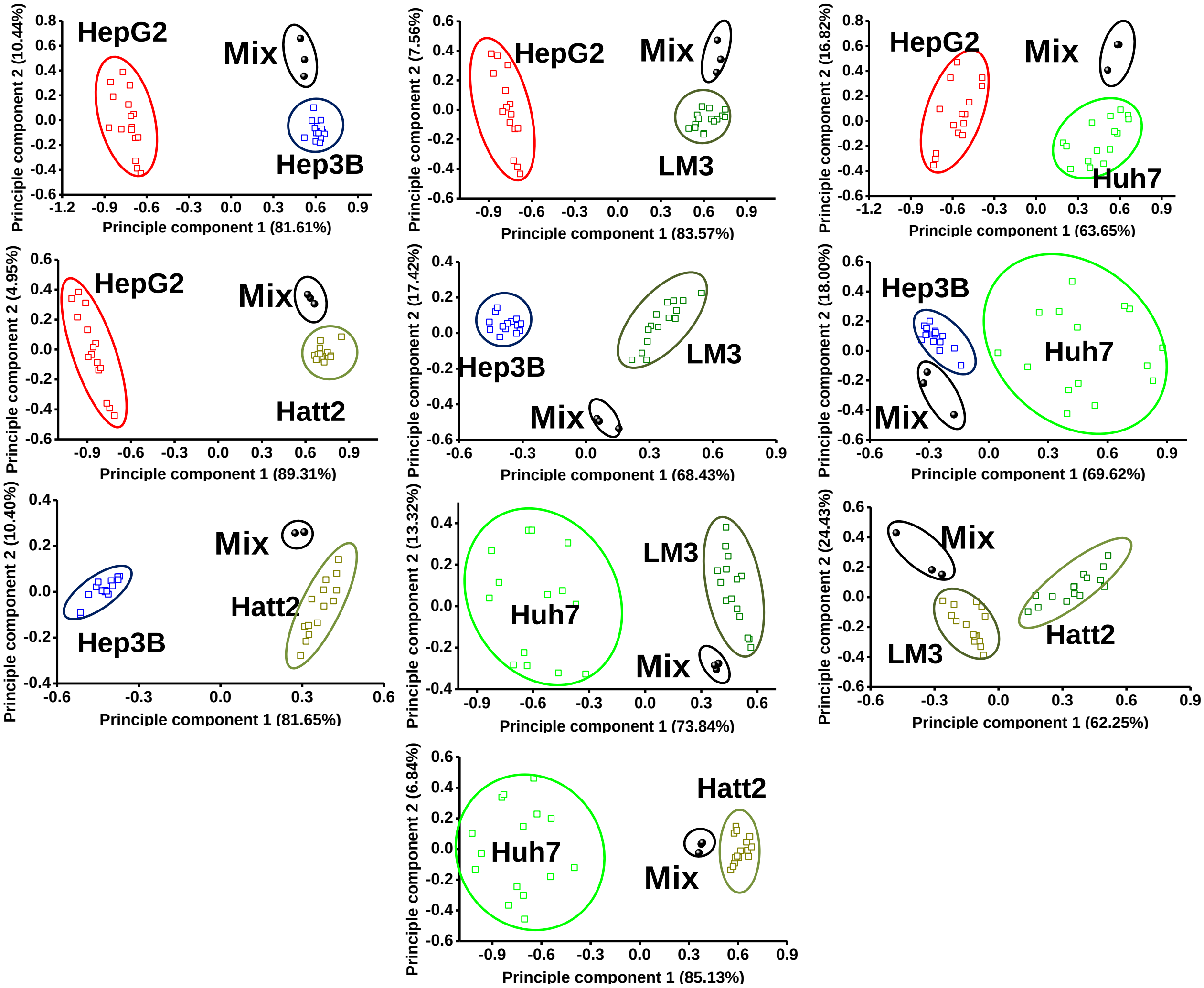

Figure S5. Principle component analysis (PCA) of cell mixture and pure cells. In each graph, "Mix" is referred to the cell mixture composed of two kinds of HCC cells with ratio =1:1. PCA process was all based on the 70 feature peaks selected by ClinproTools ($p < 0.01$). The data of pure cells used in this figure are the same with those in Figure 3b and the culture medium was DMEM.

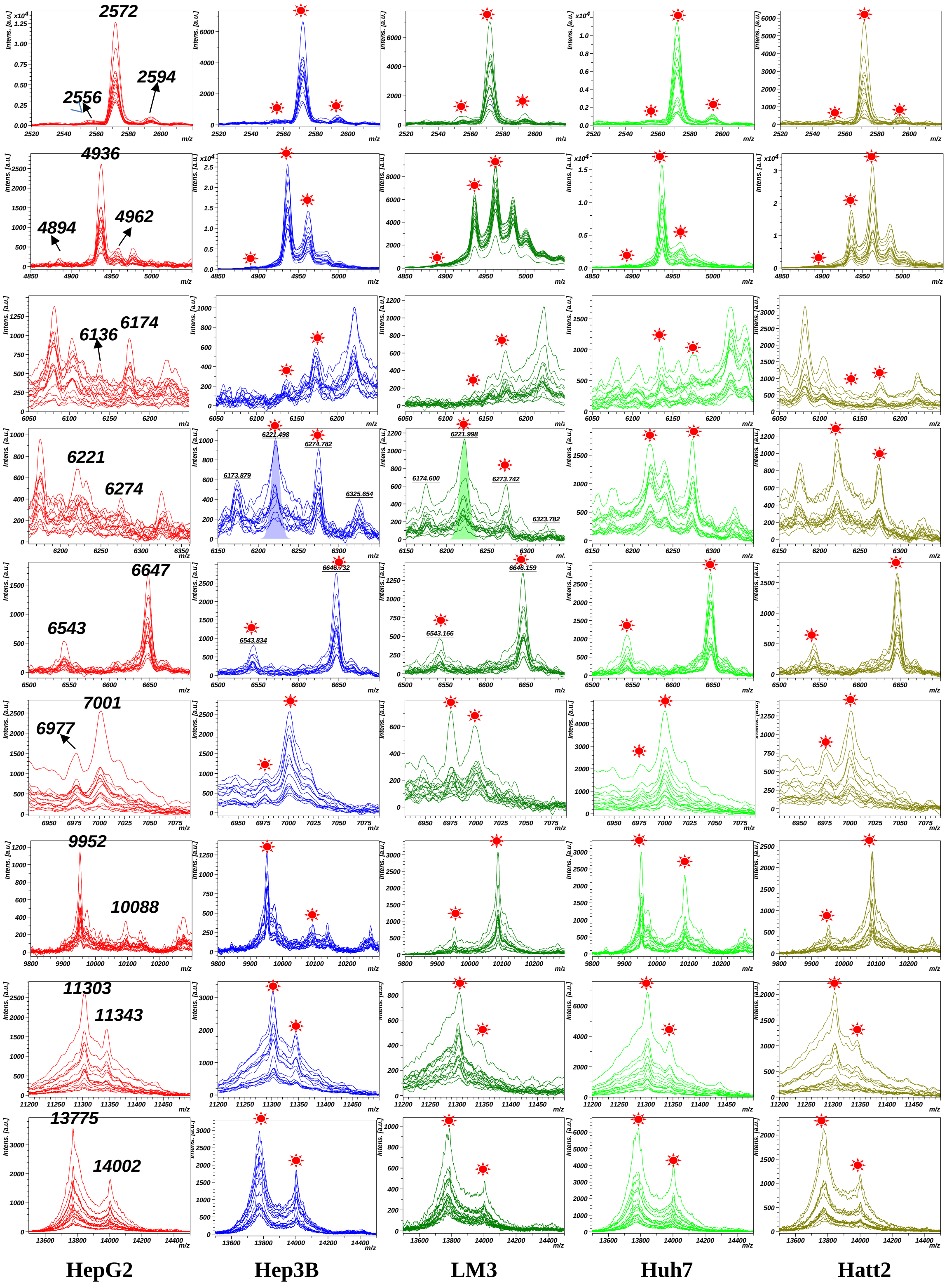

Figure S6. Magnified mass spectra of five type HCC cells at 11 pairs of dual-peaks whose RSD < 25% in each type of cells. The 11 pais of dual peaks are listed: *m/z* = 2556 vs 2572, *m/z* = 2556 vs 2594, *m/z* = 4894 vs 4962, *m/z* = 4936 vs 4962, *m/z* = 6136 vs 6174, *m/z* = 6221 vs 6274, *m/z* = 6543 vs 6647, *m/z* = 6977 vs 7001, *m/z* = 9952 vs 10088, *m/z* = 11303 vs 11343, *m/z* = 13775 vs 14002.

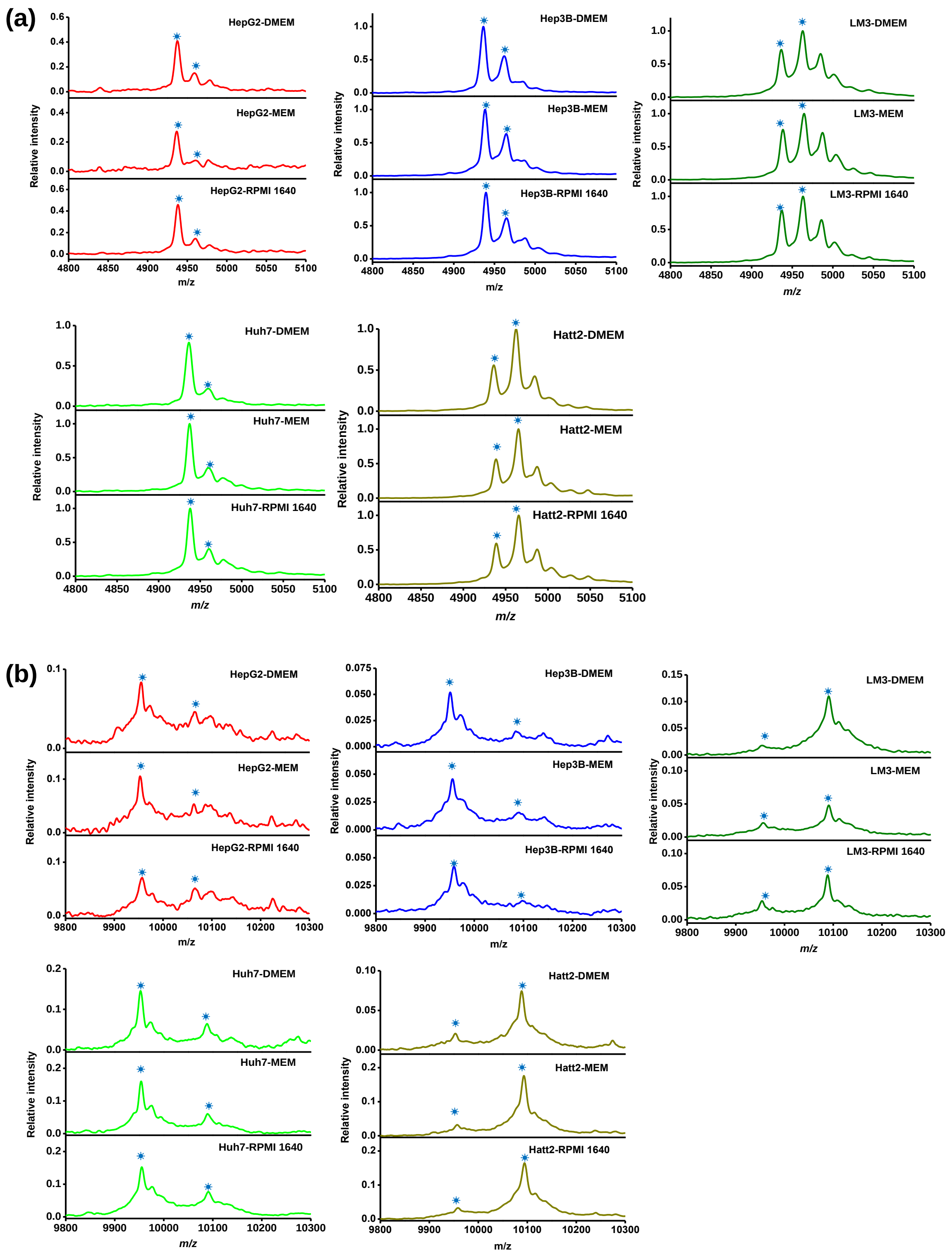

Figure S7. Magnifed mass spectra at (a) *m/z* = 4962 vs 4936, (b) *m/z* = 10088 vs 9952 of five type HCC cells cultured in three different media (DMEM, MEM and RPMI 1640). Mass spectra of cells cultured in DMEM are the same with those in Figure 2.

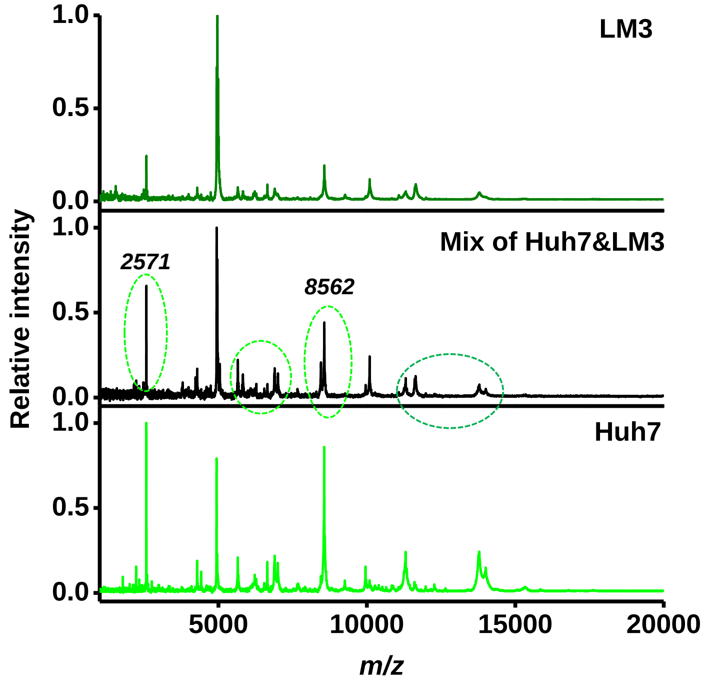

Figure S8. Typical Normalized MALDI mass spectra of (a) LM3, (b) mixture of Huh7 and LM3 with ratio =1:1 and (c) Huh7 cells cultured in DMEM. Mass spectra of Huh7 and LM3 cultured in DMEM are the same with those in Figure 2. The mass fingerprint of cell mixture seemed to overlay the spectra of Huh7 and LM3. For example, the relative intensity of the peak at *m/z* = 2571, *m/z* = 8562 in mixture sample were intermediate of those belonging to LM3 and Huh7 cells.

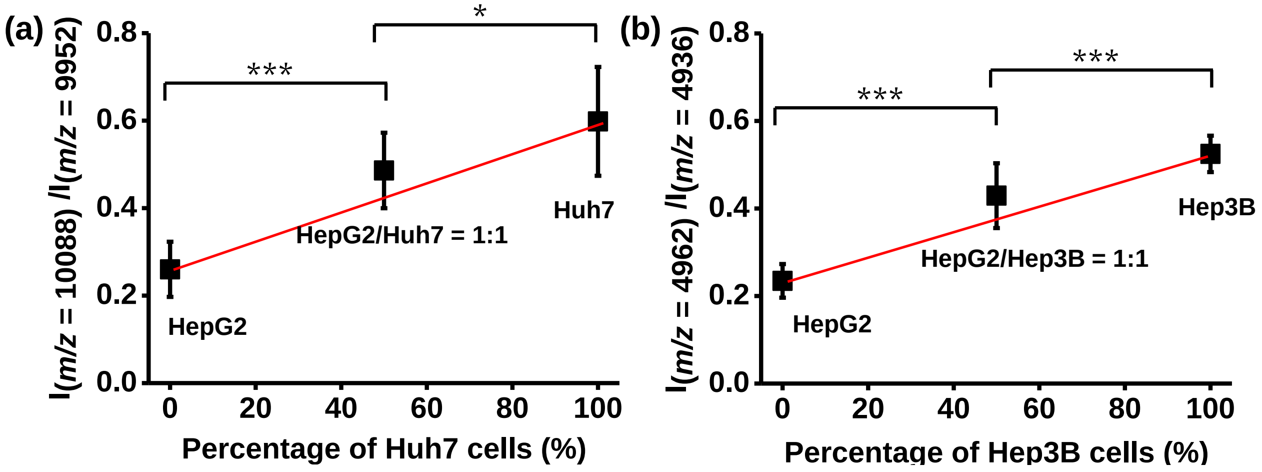

Figure S9. (a) The relationship between peak intensity ratio of *m/z* = 10088 to *m/z* = 9952 and percentage of Huh 7 cell in the cell mixture of Huh7 and HepG2 cells. (b) Relationship between peak intensity ratio of *m/z* = 4962 to *m/z* = 4936 and percentage of Hep3B cell in the cell mixture of Hep3B and HepG2. The ratio of peak pairs for pure cells was an averaged value calculated from 15 replicates while mixture was replicated for 3 tests.

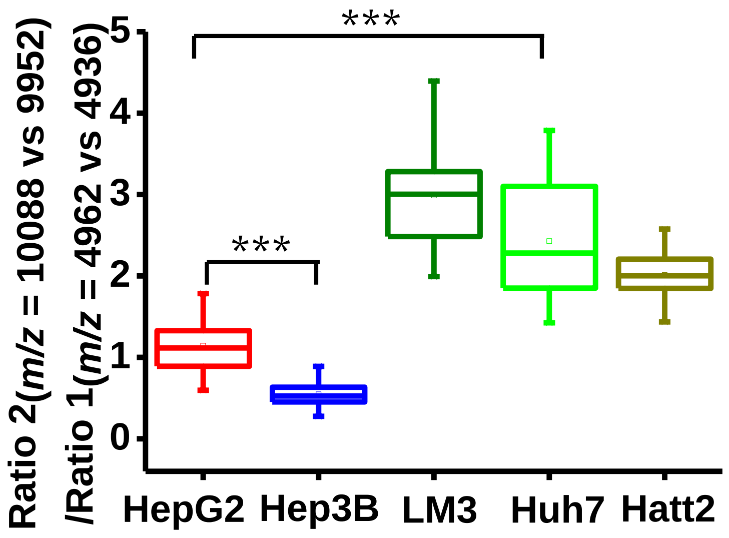

Cell type	*P* value of Ratio 2/Ratio 1
HepG2 vs Hep3B	2.04E-06
HepG2 vs LM3	7.10E-11
HepG2 vs Huh7	7.28E-07
HepG2 vs Hatt2	3.79E-07
Hep3B vs LM3	7.70E-15
Hep3B vs Huh7	7.86E-11
Hep3B vs Hatt2	7.27E-13
LM3 vs Huh7	0.027341
LM3 vs Hatt2	0.000209
Huh7 vs Hatt2	0.196562

Figure S10. Ratio of two ratio values (Ratio 1 = $I_{(m/z\,=\,4962)}$/ $I_{(m/z\,=\,4936)}$, Ratio 2 = $I_{(m/z\,=\,10088)}$/ $I_{(m/z\,=\,9952)}$) in five types of HCC cells and the *P* value results between these cells.

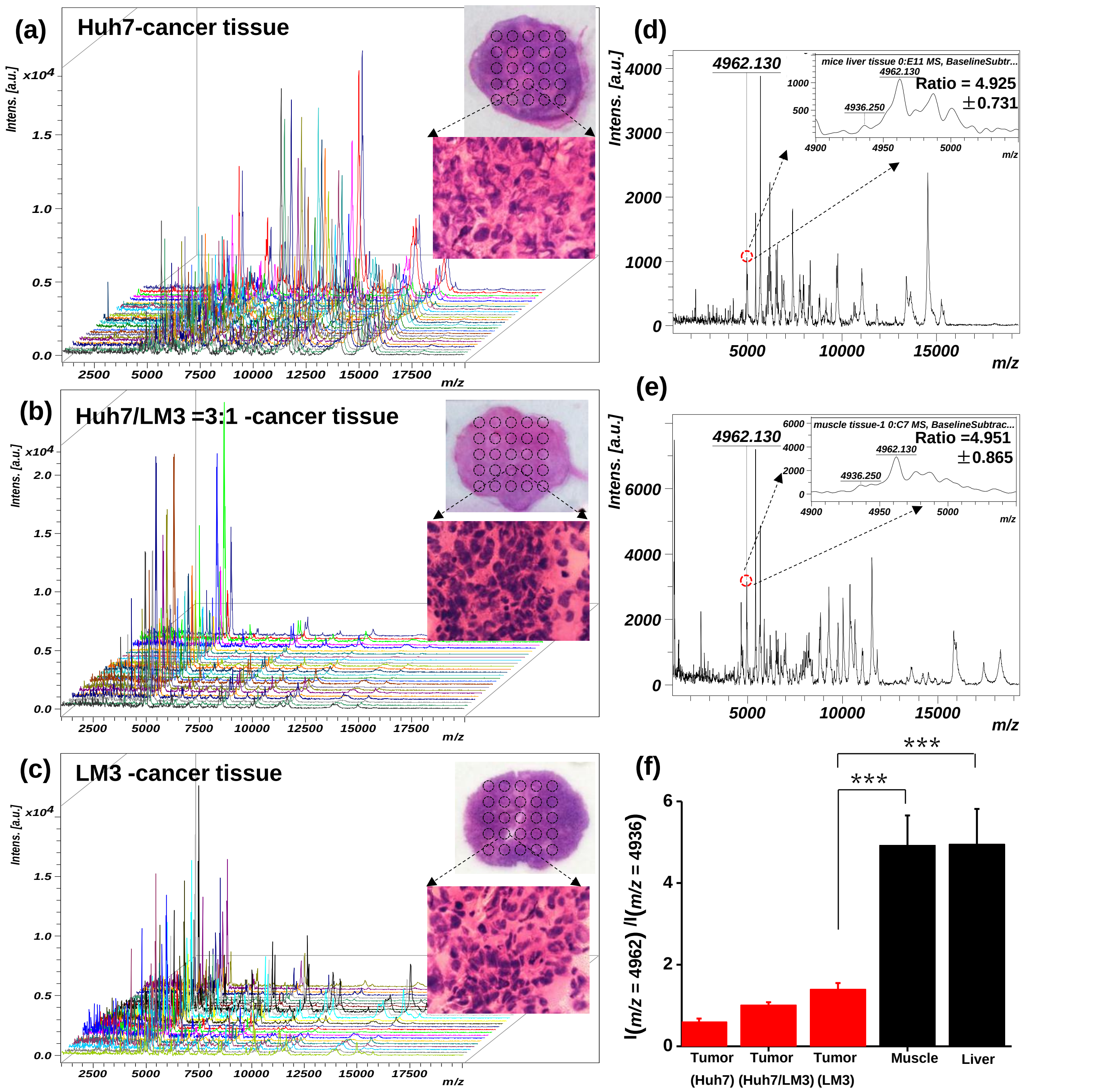

Figure S11. (a~c) MALDI mass spectra of the tissues originate from (a) pure Huh7 cells, (c) pure LM3 cells and (b) mixed cells with a ratio of Huh7/LM3 = 3:1. Insets in (a), (b), (c) are the symbolic images of the 5×5 array for MALDI detection on the tissue section which have been stained by haematoxylin and eosin for visualization. (d~e) MALDI mass spectra of (d) nude mice liver tissue and (e) nude mice muscle tissue. Insets in (d) and (e) are the magnified mass spectra at the *m/z* = 4936 and *m/z* = 4962. (f) Intensity ratio of *m/z* = 4962 to *m/z* =4936 from different tissues in the model mouse. The ratio values were calculated as $I_{(m/z = 4962)}/I_{(m/z = 4936)}$ from a 5×5 spots array on the tissues. The ratio value for mice liver tissue and mice muscle tissue was 4.925 ±0.731 and 4.951 ±0.865, respectively.

(a)

Thymosin β 4   Ac-SDKPDMAEIEKFDKSKLKKTETQEKNPLPSKETIEQEKQAGES

Thymosin β 10 Ac-ADKPDMGEIASFDKAKLKKTETQEKNTLPTKETIEQEKRSEIS

(b)

Protein name	Score	Coverage	# PSMs			
Thymosin-beta 4	94.74	90.70	33			
	Sequence	# PSMs	Modifications	MH+ [Da]	ΔM [ppm]	RT [min]
	TETQEKNPLPSKETIEQEK	11		2229.16240	16.96	20.42
	sDKPDMAEIEKFDK	14	S1(Acetyl)	1694.82529	18.30	62.36
	sDKPDMAEIEK	3	S1(Acetyl)	1304.62749	18.05	27.46
	NPLPSKETIEQEK	2		1512.81816	18.30	19.48
	NPLPSKETIEQEKQAGES	1		1985.02141	19.71	23.50
	sDKPDmAEIEKFDK	1	S1(Acetyl); M6(Oxidation)	1710.81736	16.46	34.16
	kTETQEknPLPSK	1	K1(GlyGly); K7(GlyGly); N8(Deamidated)	1728.89507	10.84	89.38
Accession	Score	Coverage	# PSMs			
Thymosin-beta 10	42.82	81.40	15			
	Sequence	# PSMs	Modifications	MH+ [Da]	ΔM [ppm]	RT [min]
	aDKPDMGEIASFDK	7	A1(Acetyl)	1565.74407	18.37	76.36
	NTLPTKETIEQEK	2		1530.82927	18.44	18.66
	NTLPTKETIEQEKR	1		1686.92795	15.29	16.42
	aDKPDmGEIASFDK	2	A1(Acetyl); M6(Oxidation)	1581.74016	18.93	44.57
	nTLPTKETIEQEK	1	N1(Deamidated)	1531.81133	17.15	22.85
	nTLPTKETIEqEK	1	N1(Deamidated); Q11(Deamidated)	1532.74578	-15.20	18.61
	kTETQEkNTLPTK	1	K1(GlyGly); K7(GlyGly)	1745.89336	-5.45	56.98

(c)

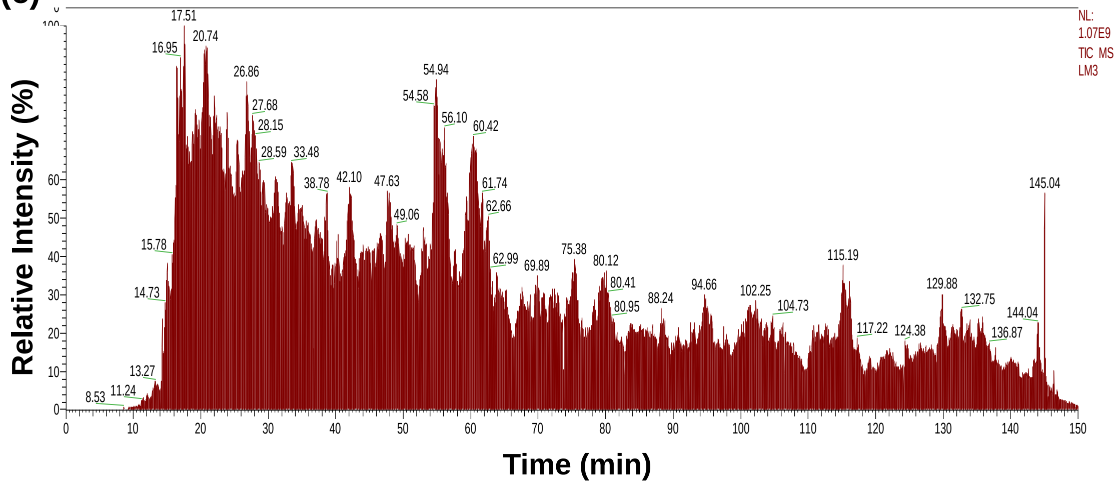

(d-1)

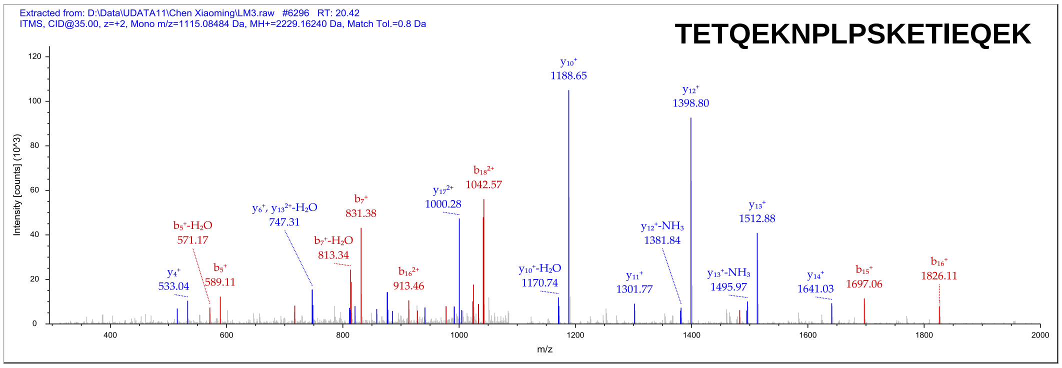
Extracted from: D:\Data\UDATA11\Chen Xiaoming\LM3.raw #6296 RT: 20.42
ITMS, CID@35.00, z=+2, Mono m/z=1115.08484 Da, MH+=2229.16240 Da, Match Tol.=0.8 Da
TETQEKNPLPSKETIEQEK
Intensity [counts] (10^3)
m/z
y_{10}^{+} 1188.65
y_{12}^{+} 1398.80
b_{18}^{2+} 1042.57
y_{17}^{2+} 1000.28
b_{7}^{+} 831.38
y_{6}^{+}, y_{13}^{2+}-H_2O 747.31
b_{5}^{+}-H_2O 571.17
b_{7}^{+}-H_2O 813.34
y_{13}^{+} 1512.88
y_{12}^{+}-NH_3 1381.84
y_{4}^{+} 533.04
b_{5}^{+} 589.11
b_{16}^{2+} 913.46
y_{10}^{+}-H_2O 1170.74
y_{11}^{+} 1301.77
y_{13}^{+}-NH_3 1495.97
y_{14}^{+} 1641.03
b_{15}^{+} 1697.06
b_{16}^{+} 1826.11

(d-2)

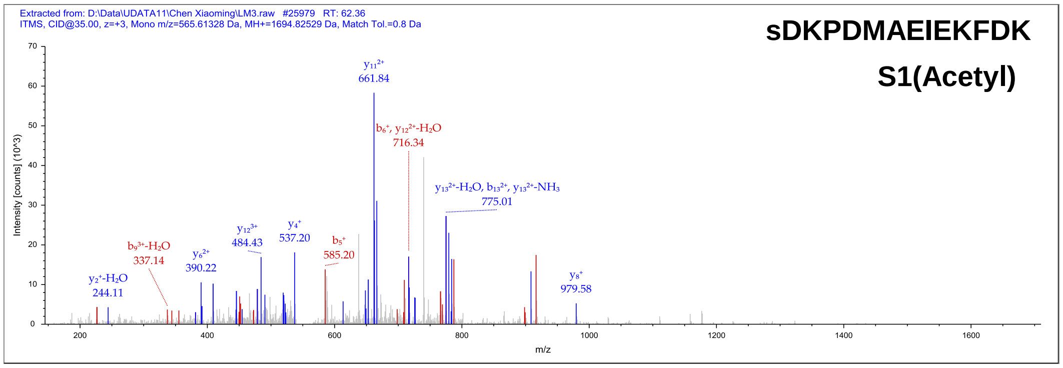
Extracted from: D:\Data\UDATA11\Chen Xiaoming\LM3.raw #25979 RT: 62.36
ITMS, CID@35.00, z=+3, Mono m/z=565.61328 Da, MH+=1694.82529 Da, Match Tol.=0.8 Da
sDKPDMAEIEKFDK
S1(Acetyl)
Intensity [counts] (10^3)
m/z
y_{11}^{2+} 661.84
b_{6}^{+}, y_{12}^{2+}-H_2O 716.34
y_{13}^{2+}-H_2O, b_{13}^{2+}, y_{13}^{2+}-NH_3 775.01
y_{12}^{3+} 484.43
y_{4}^{+} 537.20
b_{9}^{3+}-H_2O 337.14
y_{6}^{2+} 390.22
b_{5}^{+} 585.20
y_{2}^{+}-H_2O 244.11
y_{8}^{+} 979.58

(d-3)

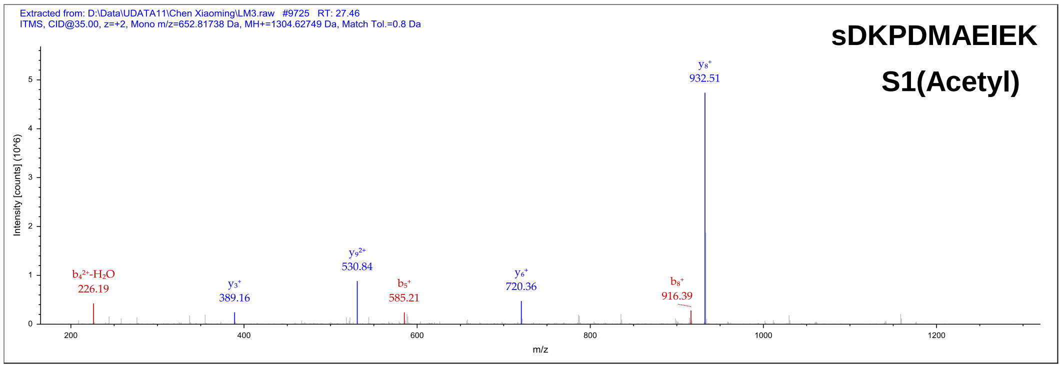
Extracted from: D:\Data\UDATA11\Chen Xiaoming\LM3.raw #9725 RT: 27.46
ITMS, CID@35.00, z=+2, Mono m/z=652.81738 Da, MH+=1304.62749 Da, Match Tol.=0.8 Da
sDKPDMAEIEK
S1(Acetyl)
Intensity [counts] (10^6)
m/z
y_{8}^{+} 932.51
y_{9}^{2+} 530.84
b_{4}^{2+}-H_2O 226.19
y_{3}^{+} 389.16
b_{5}^{+} 585.21
y_{6}^{+} 720.36
b_{8}^{+} 916.39

(d-4)

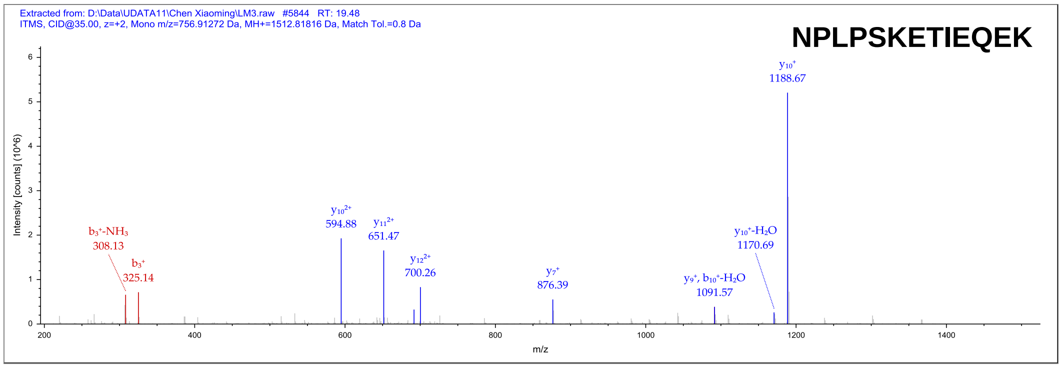
Extracted from: D:\Data\UDATA11\Chen Xiaoming\LM3.raw #5844 RT: 19.48
ITMS, CID@35.00, z=+2, Mono m/z=756.91272 Da, MH+=1512.81816 Da, Match Tol.=0.8 Da
NPLPSKETIEQEK
Intensity [counts] (10^6)
m/z
y_{10}^{+} 1188.67
y_{10}^{2+} 594.88
y_{11}^{2+} 651.47
b_{3}^{+}-NH_3 308.13
b_{3}^{+} 325.14
y_{12}^{2+} 700.26
y_{7}^{+} 876.39
y_{10}^{+}-H_2O 1170.69
y_{9}^{+}, b_{10}^{+}-H_2O 1091.57

(e-1)

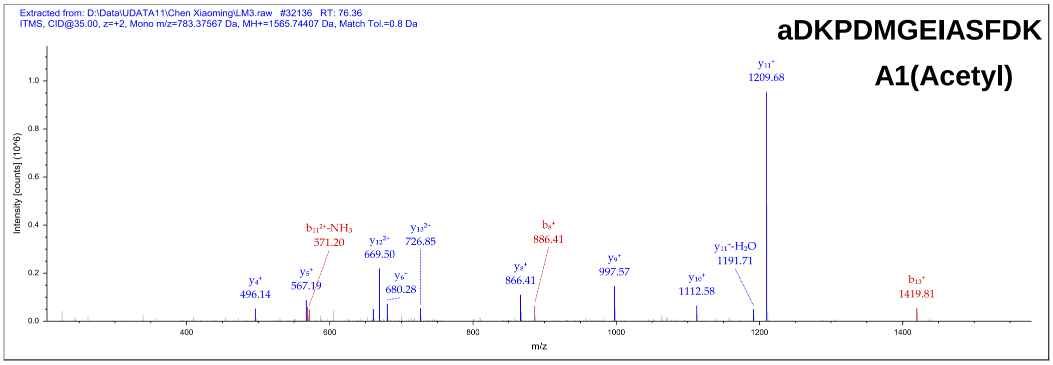

(e-2)

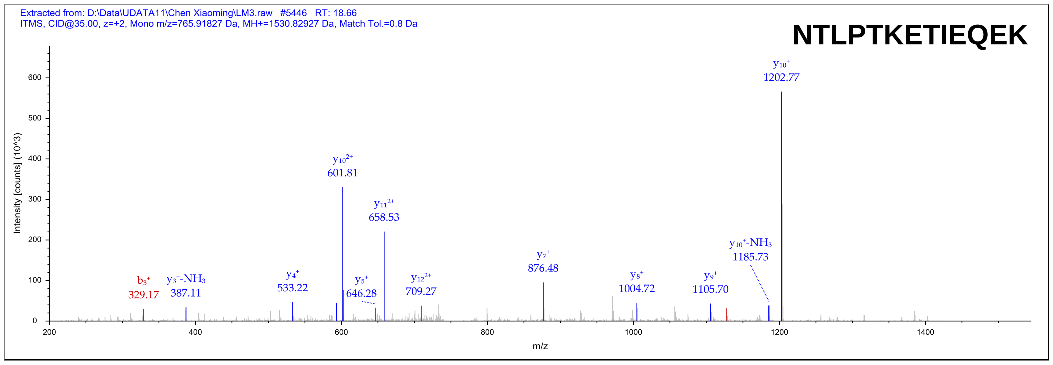

(e-3)

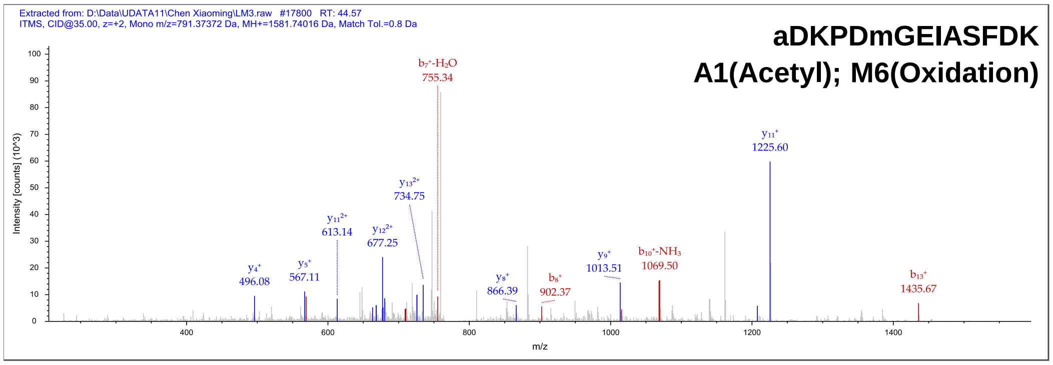

Figure S12. LC-MS/MS identification results of thymosin β4 and thymosin β10. (a) Amino acid sequence of thymosin β4 and thymosin β10. (b) Information of the peptide sequence identified by LC-MS/MS. (c) Total ion current spectrum of the LM3 cell sample after trypsin lysis procedure. (d-e) MS/MS spectra of the identified peptide sequence belonging to (d) thymosin β4 and (e) thymosin β10 with high PSMs, respectively.

Table S1. Normalized intensity of 70 feature peaks from the mass spectra of five types of HCC cells selected by ClinproTools software

	Normalized Intensity (Average value ±Error)				
**m/z of 70 feature peaks**	**HepG2**	**Hep3B**	**LM3**	**Huh7**	**Hatt2**
**1099.13**	0.129±0.029	0.058±0.022	0.01±0.011	0.048±0.023	0.029±0.016
**1779.04**	0.052±0.018	0.018±0.009	0.012±0.014	0.046±0.024	0.005±0.007
**2006.99**	0.058±0.02	0.006±0.007	0.019±0.013	0.023±0.017	0.007±0.007
**2154.19**	0.129±0.027	0.012±0.006	0.04±0.018	0.041±0.018	0.017±0.007
**2227.23**	/	/	0.009±0.01	0.066±0.044	0.004±0.006
**2328.43**	0.075±0.011	0.019±0.004	0.043±0.017	0.053±0.019	0.017±0.008
**2349.52**	0.033±0.007	0.007±0.005	0.026±0.016	0.02±0.014	0.018±0.005
**2467.37**	0.009±0.014	0.033±0.007	0.023±0.015	0.038±0.006	0.013±0.007
**2482.81**	0.019±0.016	0.022±0.01	0.029±0.024	0.019±0.012	0.022±0.008
**2555.58**	0.062±0.012	0.012±0.007	0.041±0.019	0.043±0.012	0.012±0.009
**2571.90**	1±0	0.221±0.048	0.508±0.191	0.658±0.26	0.132±0.065
**2593.84**	0.11±0.019	0.023±0.006	0.053±0.023	0.073±0.033	0.017±0.011
**2634.26**	0.07±0.023	0.001±0.003	0.001±0.005	0.016±0.015	0.002±0.004
**2754.26**	0.031±0.016	0.008±0.005	0.021±0.013	0.035±0.017	0.011±0.008
**2775.74**	0.005±0.01	0.01±0.007	0.011±0.014	0.012±0.011	0.031±0.011
**2978.37**	/	0.03±0.006	0.002±0.007	0.024±0.016	0.109±0.035
**3788.64**	0.028±0.013	0.001±0.002	0.015±0.013	0.024±0.012	0.001±0.004
**3838.72**	0.005±0.01	0.013±0.008	0.024±0.016	/	0.019±0.004
**3937.48**	0.015±0.013	0.021±0.01	0.004±0.008	0.016±0.015	0.006±0.006
**4213.78**	0.019±0.027	0.023±0.01	0.029±0.016	0.003±0.007	0.04±0.006
**4225.05**	0.1±0.041	0.017±0.009	0.023±0.017	0.033±0.013	0.033±0.004
**4281.40**	0.104±0.03	0.097±0.017	0.079±0.011	0.157±0.023	0.036±0.006
**4418.30**	0.119±0.038	0.047±0.006	0.064±0.025	0.121±0.029	0.057±0.009
**4735.73**	0.05±0.019	0.033±0.007	0.049±0.01	0.039±0.017	0.038±0.008
**4894.11**	0.003±0.007	0.029±0.009	0.038±0.006	0.035±0.011	0.021±0.014
**4936.25**	0.194±0.053	1±0	0.711±0.024	0.972±0.066	0.588±0.045
**4962.13**	0.045±0.013	0.525±0.041	1±0	0.244±0.026	1±0
**4983.82**	0.027±0.02	0.144±0.062	0.403±0.042	0.121±0.013	0.467±0.106
**4999.71**	0.007±0.011	0.062±0.034	0.163±0.052	0.049±0.026	0.195±0.052
**5044.08**	0.002±0.007	0.007±0.012	0.058±0.011	0.032±0.017	0.06±0.015
**5068.20**	0.012±0.014	0.006±0.01	0.02±0.017	0.022±0.013	0.027±0.016
**5419.21**	0.043±0.013	0.016±0.008	/	0.01±0.011	0.001±0.002

**5608.56**	0.036±0.01	0.021±0.008	0.02±0.012	0.028±0.02	0.008±0.006
**5650.12**	0.192±0.063	0.103±0.03	0.08±0.032	0.261±0.107	0.05±0.024
**5670.59**	0.094±0.041	0.054±0.026	0.014±0.022	0.123±0.06	0.026±0.015
**5991.42**	0.055±0.028	0.014±0.007	0.015±0.008	0.023±0.011	0.014±0.005
**6080.82**	0.123±0.048	0.001±0.003	/	0.033±0.022	0.086±0.018
**6104.03**	0.08±0.045	/	/	0.023±0.02	0.051±0.014
**6136.22**	0.055±0.023	0.008±0.005	0.003±0.006	0.049±0.017	0.015±0.01
**6174.32**	0.077±0.024	0.025±0.006	0.029±0.014	0.047±0.02	0.03±0.006
**6221.52**	0.058±0.024	0.032±0.01	0.052±0.021	0.113±0.017	0.039±0.008
**6274.02**	0.026±0.022	0.03±0.007	0.033±0.008	0.089±0.021	0.031±0.01
**6325.26**	0.033±0.017	0.013±0.005	0.001±0.002	0.029±0.017	0.007±0.006
**6543.21**	0.03±0.019	0.024±0.007	0.022±0.015	0.053±0.015	0.017±0.006
**6646.69**	0.149±0.072	0.079±0.025	0.088±0.019	0.187±0.038	0.059±0.012
**6889.47**	0.185±0.079	0.1±0.036	0.053±0.021	0.248±0.107	0.046±0.022
**6947.02**	0.059±0.042	0.029±0.019	0.018±0.017	0.08±0.044	0.017±0.012
**6976.82**	0.101±0.029	0.039±0.01	0.027±0.016	0.102±0.032	0.023±0.01
**7000.97**	0.139±0.058	0.081±0.034	0.038±0.019	0.2±0.085	0.033±0.021
**7920.40**	0.024±0.012	0.017±0.016	0.003±0.006	0.036±0.034	/
**8448.24**	0.429±0.187	0.047±0.006	0.09±0.016	0.108±0.017	0.06±0.012
**8469.44**	0.165±0.091	0.031±0.005	0.053±0.018	0.061±0.018	0.035±0.009
**8562.24**	0.476±0.138	0.451±0.028	0.506±0.056	0.795±0.083	0.207±0.025
**9513.30**	0.013±0.011	/	/	0.013±0.014	0±0.001
**9743.44**	0.032±0.013	/	/	0.004±0.007	/
**9952.93**	0.079±0.02	0.047±0.009	0.04±0.004	0.141±0.028	0.02±0.003
**10088.74**	0.02±0.007	0.013±0.003	0.166±0.029	0.086±0.03	0.075±0.024
**10274.70**	0.022±0.014	0.01±0.004	0.014±0.008	0.033±0.009	0.011±0.003
**10396.53**	0.036±0.013	/	0.013±0.004	0.027±0.011	/
**10517.59**	0.034±0.008	0.02±0.003	0.01±0.006	0.012±0.011	0.003±0.003
**10838.80**	0.078±0.026	0.034±0.01	0.015±0.007	0.04±0.015	0.007±0.003
**10885.67**	0.047±0.012	0.012±0.005	/	0.032±0.012	0.004±0.003
**11069.65**	0.004±0.015	/	0.049±0.014	0.029±0.01	0.022±0.009
**11302.87**	0.151±0.079	0.099±0.041	0.061±0.029	0.273±0.128	0.049±0.029
**11343.04**	0.095±0.045	0.061±0.031	0.021±0.018	0.147±0.07	0.029±0.015
**11603.55**	0.038±0.012	0.015±0.004	0.143±0.016	0.051±0.012	0.031±0.007
**11981.62**	0.03±0.009	0.027±0.007	0.017±0.004	0.031±0.006	0.005±0.003
**12273.26**	0.021±0.012	0.007±0.001	0.001±0.004	0.048±0.014	0.008±0.002
**13774.69**	0.208±0.101	0.099±0.038	0.062±0.024	0.259±0.103	0.058±0.027

**14002.40**	0.1±0.05	0.061±0.027	0.031±0.014	0.162±0.073	0.031±0.016

Table S2. *P* values calculated with Student's t test of the 11 pair dual-peaks between each two type of five types of HCC cells

	P values of 11 pair dual-peaks										
**Cell mixture type**	**2556 vs 2572**	**2556 vs 2594**	**4894 vs 4962**	**4936 vs 4962**	**6136 vs 6174**	**6221 vs 6274**	**6543 vs 6647**	**6977 vs 7001**	**9952 vs 10088**	**11303 vs 11343**	**13774 vs 14002**
**HepG2 vs Hep3B**	7.935E-04	8.067E-01	1.678E-13	4.786E-18	4.244E-12	1.333E-05	3.905E-03	2.235E-06	3.832E-01	9.065E-02	1.844E-08
**HepG2 vs LM3**	1.306E-05	4.270E-01	2.447E-19	1.066E-33	4.913E-15	7.557E-02	8.276E-04	5.518E-03	7.928E-17	2.338E-08	9.494E-02
**HepG2 vs Huh7**	1.744E-01	9.702E-01	3.176E-07	1.509E-01	2.068E-04	4.547E-04	5.016E-02	4.457E-05	3.734E-10	7.508E-06	1.241E-09
**HepG2 vs Hatt2**	5.130E-05	8.164E-03	1.521E-10	4.556E-27	9.218E-01	8.300E-04	3.143E-02	6.704E-01	6.475E-17	5.298E-01	1.254E-20
**Hep3B vs LM3**	2.209E-01	6.989E-01	2.741E-07	6.911E-30	1.408E-03	1.089E-05	6.198E-01	2.561E-10	4.963E-08	1.211E-06	3.656E-06
**Hep3B vs Huh7**	8.361E-02	7.792E-01	2.372E-11	4.250E-19	6.016E-12	1.134E-02	2.063E-01	3.019E-01	5.243E-04	3.452E-03	4.539E-01
**Hep3B vs Hatt2**	2.500E-01	1.093E-02	8.543E-05	2.267E-24	2.264E-10	4.523E-03	3.095E-01	1.982E-05	1.873E-07	2.852E-01	1.941E-18
**LM3 vs Huh7**	7.640E-03	3.878E-01	5.905E-18	4.575E-35	3.485E-14	3.061E-04	6.889E-02	4.774E-09	2.146E-03	2.961E-05	2.769E-07
**LM3 vs Hatt2**	9.166E-01	1.630E-03	1.368E-01	2.799E-09	2.351E-13	7.588E-04	1.180E-01	3.748E-03	1.005E-03	1.215E-07	4.921E-20
**Huh7 vs Hatt2**	1.141E-02	7.690E-03	1.280E-09	3.229E-27	5.819E-04	6.076E-01	7.977E-01	3.282E-04	1.059E-05	1.040E-04	2.436E-18

$P < 0.01$ means the significant difference between cell types.

Table S3. Feature peaks information ranking first in the principle component analysis

m/z of feature peaks	Coefficient of the peaks in p1	m/z of feature peaks	Coefficient of the peaks in p2
**4962.13**	**0.59554**	**4936.25**	**0.750937**
2571.90	-0.5483	8562.24	0.374808
4983.82	0.265733	2571.90	-0.22771
4936.25	0.205614	4962.13	-0.22539
8562.24	-0.19411	8448.24	-0.18991
8448.24	-0.18672	4983.82	-0.15158
6889.47	-0.1386	11302.87	0.144088
4999.71	0.120251	5650.12	0.104848
5650.12	-0.11933	6889.47	0.095461
13774.69	-0.11789	7000.97	0.095435
